# A real-world study of wearable sensors in Parkinson’s disease

**DOI:** 10.1038/s41531-021-00248-w

**Published:** 2021-11-29

**Authors:** Jamie L. Adams, Karthik Dinesh, Christopher W. Snyder, Mulin Xiong, Christopher G. Tarolli, Saloni Sharma, E. Ray Dorsey, Gaurav Sharma

**Affiliations:** 1 Department of Neurology, University of Rochester Medical Center, Rochester, NY USA; 2 Center for Health + Technology, University of Rochester Medical Center, Rochester, NY USA; 3 Department of Electrical and Computer Engineering, University of Rochester, Rochester, NY USA; 4 Rochester General Hospital, Microbiology, Rochester, NY USA; 5 Michigan State University College of Human Medicine, East Lansing, MI USA

**Keywords:** Neurological manifestations, Parkinson's disease

## Abstract

Most wearable sensor studies in Parkinson’s disease have been conducted in the clinic and thus may not be a true representation of everyday symptoms and symptom variation. Our goal was to measure activity, gait, and tremor using wearable sensors inside and outside the clinic. In this observational study, we assessed motor features using wearable sensors developed by MC10, Inc. Participants wore five sensors, one on each limb and on the trunk, during an in-person clinic visit and for two days thereafter. Using the accelerometer data from the sensors, activity states (lying, sitting, standing, walking) were determined and steps per day were also computed by aggregating over 2 s walking intervals. For non-walking periods, tremor durations were identified that had a characteristic frequency between 3 and 10 Hz. We analyzed data from 17 individuals with Parkinson’s disease and 17 age-matched controls over an average 45.4 h of sensor wear. Individuals with Parkinson’s walked significantly less (median [inter-quartile range]: 4980 [2835–7163] steps/day) than controls (7367 [5106–8928] steps/day; *P* = 0.04). Tremor was present for 1.6 [0.4–5.9] hours (median [range]) per day in most-affected hands (MDS-UPDRS 3.17a or 3.17b = 1–4) of individuals with Parkinson’s, which was significantly higher than the 0.5 [0.3–2.3] hours per day in less-affected hands (MDS-UPDRS 3.17a or 3.17b = 0). These results, which require replication in larger cohorts, advance our understanding of the manifestations of Parkinson’s in real-world settings.

## Introduction

Parkinson’s disease (PD) is the world’s fast-growing neurological disorder^1^ and results in motor^2^, cognitive^3^, psychiatric^4^, and non-motor symptoms. Current means of assessing PD are largely limited to episodic in-person assessments conducted in the clinic. However, due to intra- and inter-day symptom fluctuations within an individual and wide variability in disease characteristics across individuals, collecting and assessing the data outside the clinic environment is essential. Analyzing real-world data^5–7^ can help us understand the natural history of the disease, longitudinal progression, and efficacy of new treatments in PD.

Wearable sensors have seen increasing use over the past decade for measuring different motor features of PD^8–15^. Wearable sensors can provide continuous, objective, and longitudinal data in both clinical and real-world settings. Several studies have used wearable sensors to perform activity, gait, and motor assessments in PD^16–19^. However, most studies to date have focused on in-clinic assessments leaving the real-world experience of the patient unexamined. Although some studies have reported real-world data assessments, only a few^20–22^ have systematically assessed tremor prevalence and impact. In this study we attempt to answer several fundamental questions such as: what proportion of a day do individuals with PD experience tremor?, what is the variation in tremor amplitude and frequency over the course of a day?, and how do these relate to the participant’s activities^23^. To effectively summarize the fine-grained variations over time revealed by the wearable sensor data, we also present a useful clock-based visualization that allows physicians, researchers, and patients to readily understand and interpret the results.

In this observational study, our goal was to examine the activity profile and subsequently analyze the gait and tremor characteristics of participants in the clinic and real-world using wearable sensors.

## Results

### Study population

Twenty individuals with PD and 22 controls were enrolled in the study. Three individuals with PD were excluded from the analysis due to sensor problems. Five controls were excluded from analysis after age-matching participants with PD to the controls. Table 1 provides the characteristics of the study participants. Data from 17 PD (mean [standard deviation] age: 66.4 [11.3] years; 41.2% women) and 17 control (64.0 [9.9] years; 76.5% women) participants were used for analysis. The methodology of Stebbins et al. ^24^ classified 8 of the PD participants as postural instability/gait difficulty (PIGD), 7 as tremor dominant (TD), and 2 as indeterminate motor phenotypes.

**Table 1:** Characteristics of the study population.

Characteristic	Parkinson’s disease (*n* = 17)	Controls (*n* = 17)
Demographic		
Age, mean [standard deviation]	66.4 [11.3]	64.0 [9.9]
Sex, women %	41.2	76.5
Ethnicity, white %	100	100
Hispanic ethnicity %	0.0	0.0
Education, 4-year college degree or higher %	94.1	47.1
Currently employed or student %	17.6	41.2
Currently married or in a domestic partnership %	94.1	82.4
Clinical		
Hoehn & Yahr stage	1.9 [0.8]	N/A
Years since diagnosis, mean [standard deviation]	4.8 [4.0]	N/A
Montreal Cognitive Assessment score (0–30)^b^, mean [standard deviation]	27.2 [2.1]	27.9 [1.5]
MDS-UPDRS—total rest tremor score (3.17a–3.17e), mean [standard deviation], range	2.0 [1.6], 0.0–4.0	N/A
MDS-UPDRS—total motor score (0–132)^a^, mean [standard deviation]	20.9 [7.9]	2.2 [2.1]
Timed Up and Go, mean [standard deviation] s	10.4 [2.6]	8.4 [1.1]
10-m Walk Test, mean [standard deviation] s	4.7 [1.1]	4.1 [0.5]

Values are mean [standard deviation] unless otherwise noted. MDS-UPDRS, Movement Disorder Society-Unified Parkinson’s Disease Rating Scale; N/A not applicable.

^a^Higher score indicates greater disability.

^b^Higher score indicates greater cognitive function.

### Activity analysis

**Fig. 1:**
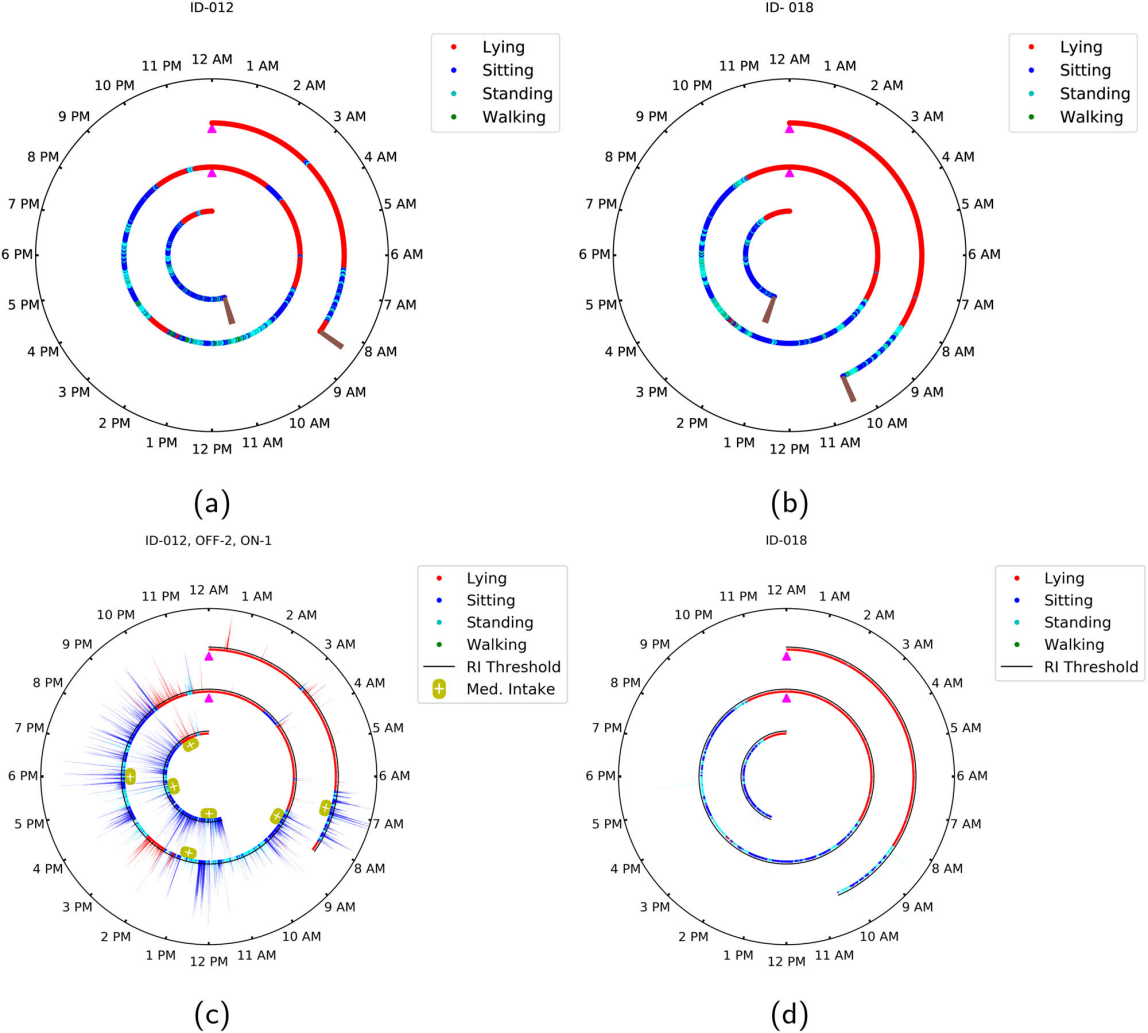
Clock visualization of activity and tremor for a PD and a control participant. A 24-h clock format visualization for **a** activity for a participant with PD and **b** activity for a control participant, **c** activity, tremor, and medication for a participant with PD, and **d** activity and (lack of) tremor for a control participant. Data over the duration of sensor wear is depicted in the polar plots, with the brown marker in the innermost and the outermost circle representing the start and end of the sensor wear duration, respectively. The concentric circles each represent different days and the magenta markers (located at 12 AM position) indicate the transition from one calendar day to the next. The activity is classified into one of four classes (lying, sitting, standing, and walking) for each 2-second interval and represented as a corresponding color-coded dot in the polar plot. Each color-coded bar on the polar plots in (**c**) and (**d**) jointly represent the rhythmicity index and activity state over a 2-s interval, with the color identifying the activity state and the height of the bar indicating the rhythmicity index (tremor amplitude). The black circle above each radius represents the rhythmicity index threshold, which is set to a value of 3.3. The yellow capsule-shaped markers below each radius represent the medication intake timings for the participants with PD.

The proportion of time spent per day for different activities for participants with PD and controls is reported in Table 2. In comparison with the controls, individuals with PD spent less time walking and similar amounts of time lying down and sitting. A sample of the activity patterns of the participants over the full duration of their sensor wear is illustrated in Fig. 1a (for an individual with PD) and Fig. 1b (for a control participant) using a clock-based motif for visualization. The observations from the activity clocks were consistent with the self-reported activity logs of both participants.

**Table 2:** Comparison of activity and gait parameters for participants with and without Parkinson’s disease.

Motor features	Parkinson’s disease, median [interquartile range]	Control, median [interquartile range]	*P*-value
Activity metrics			
Lying proportion, h/day	9.1 [8.1–9.9]	8.3 [8.0–9.8]	*P* = 0.65
Sitting proportion, h/day	10.7 [9.7–11.6]	10.3 [8.9–12.6]	*P* = 0.73
Standing proportion, h/day	3.3 [2.5–4.3]	3.6 [2.6–4.0]	*P* = 0.47
Walking proportion, h/day	0.9 [0.5–1.3]	1.4 [0.9–1.6]	*P* = 0.04
Gait metrics			
Steps per day	4980 [2835–7163]	7367 [5106–8928]	*P* = 0.04
Step length, m	0.52 [0.50–0.55]	0.54 [0.50–0.55]	*P* = 0.28
Gait speed, m/s	0.91 [0.88–0.98]	0.92 [0.86–0.97]	*P* = 0.47
Step duration, s/step	0.58 [0.56–0.59]	0.58 [0.57–0.59]	*P* = 0.22
Step co-ordination	0.25 [0.24–0.27]	0.30 [0.26–0.34]	*P* = 0.01

### Gait analysis

The gait parameters for individuals with PD and for controls are reported in Table 2. Individuals with PD took significantly fewer steps (median [interquartile range]: 4980 [2835–7163] steps/day) than controls (7367 [5106–8928] steps/day; *P* = 0.04). While step duration was similar to that of controls, step length and gait speed were slightly lower for PD than controls. Cross-correlation analysis quantified the inter-leg coordination, which was lower for individuals with PD than controls.

### Tremor analysis

The tremor proportion in the sitting and standing states, for most-affected and less-affected hands among those with PD, and the right hand for control participants, is illustrated in the Supplementary Fig. 1. The median tremor proportion per day for most-affected hands of the participants with PD was (median [range]: 6.5 [1.5–24.6] % or 1.6 [0.4–5.9] h) higher than that of less-affected hands of the participants with PD (2.2 [1.4–9.4]% or 0.5 [0.3–2.3] h; *P* = 0.003) and the right hand of unaffected controls (1.6 [1.1–2.5]% or 0.4 [0.3–0.6] h; *P* < 0.001). Also, less-affected hands of participants with PD (defined as MDS-UPDRS 3.17a or 3.17b = 0), had a higher tremor proportion in comparison with the right hand of the control participants (*P*﻿ = 0.003). The multiple comparison analysis results are provided in the Supplementary Note 1. We also observed that the median tremor proportion per day for most-affected hands of the participants with TD motor phenotype was (median [range]: 17.9 [2.5–24.6]% or 4.3 [0.6–5.9] h) significantly higher than that of the participants with PIGD motor phenotype (2.7 [1.5–4.1]% or 0.7 [0.4–1.0] h; *P* = 0.004). There was a strong correlation between the proportion of time with tremor and the MDS-UPDRS maximal at-rest tremor score for both OFF (right hand: *r* = 0.79; *P* = 0.002; left hand: *r* = 0.48; *P* = 0.11) and ON (right hand: *r* = 0.50; *P* = 0.04; left hand: *r* = 0.45; *P* = 0.07) assessments. The real-world tremor proportion correlated strongly with the in-clinic tremor proportion reported on the MDS-UPDRS (right hand: *r* = 0.88; *P* < 0.001; left hand: *r* = 0.87; *P* < 0.001) among participants with PD. We observed a strong and statistically significant correlation between most-affected hand tremor proportion and constancy of the rest tremor (MDS-UPDRS 3.18 ON assessment) (*r* = 0.84 and *P* < 0.001).

To examine the variation in hand tremor amplitude over the course of sensor wear, we augmented the activity clock to also depict the variation in rhythmicity index, which characterizes the intensity of quasi-periodic movements characteristic of tremor recorded by the sensors (see Methods section). As an example, rhythmicity index profiles for the same participants (described previously in Fig. 1a, b) with and without PD are shown in Fig. 1c, d, respectively. In general, the magnitude of the rhythmicity index is highest for the sitting intervals, followed by the standing intervals, and then lying down intervals, which have the lowest values of the rhythmicity index. Note that the lying down intervals in our analysis comprised both sleeping and non-sleeping intervals. Control participants exhibited a very low rhythmicity index, typically below the threshold (threshold value = 3.3), as exemplified in Fig. 1d. Additional clock visualizations for other participants with and without PD are included in the Supplementary Material (Supplementary Figs. 2–4). A scatter plot and heatmap illustration showing the joint behavior of rhythmicity index and peak frequency for participants with and without PD are included in the Supplementary Material (Supplementary Figs. 5 and 6).

## Discussion

Wearable sensors are capable of measuring key motor features of PD outside the clinic. In this observational study, we assessed motor characteristics for participants with PD using wearable sensors in both clinical and real-world settings. Combined with intuitive and interpretable visualization, such as the 24-h clock format that we introduced, these data and analyses provide new insights into the lives of individuals with PD. Analysis of gait activity revealed that individuals with PD walked less than the controls—a finding that has also been reported in prior studies. One of the studies^17^, which monitored 220 individuals with PD over approximately 100 days, found that, on average [standard deviation], they walked 72 [39] min per day, which was similar to 60 [32] min per day in our study of 17 participants with PD.

We also assessed important gait features such as steps per day, gait speed, step duration, and step coordination. Gait analysis revealed that, compared with controls, the participants with PD had similar step duration, step length, and walk speed, and poorer coordination between the two legs. Gait speed, considered as the sixth vital sign^25,26^, is an important indicator of survival in older adults^27,28^. Few studies have analyzed gait speed in PD for real-world settings. While one study^29^ reported an average [standard deviation] gait speed of 0.83 [0.16] m/s, slightly smaller than our study (0.91 [0.07] m/s), another study^30^ estimated the average gait speed of 0.66 [0.14] m/s. The same study^30^ estimated the average [standard deviation] steps per day to be 4099 [2673] steps/day, which was lower than the 5650 [3331] steps/day that we observed in our study. The demographic data of this study^30^ which reported a lower gait speed revealed that individuals had more advanced PD (average Hoehn and Yahr^31^ stage of 2.8 and baseline MDS-UPDRS motor score of 34.8) in comparison with our study (average Hoehn and Yahr stage of 1.9 and baseline MDS-UPDRS motor score of 20.9), which may explain the different findings. Table 3 provides a comparison of steps per day and gait speed reported in recent studies.

**Table 3:** Comparison of steps per day and gait speed for participants with Parkinson’s disease from recent observational studies from 2004 to 2019.

Study	Sample size	Age, years	Disease duration, years	Mean Hoehn & Yahr stage	Mean [SD] steps per day	Mean [SD] gait speed, m/s
Current study	17	66.4 [11.3]	4.8 [4.0]	1.9	5650 [3331]	0.91 [0.07]
Pradhan et al. ^46^, 2019	30	68.6 [5.9]	7.8 [5.0]	1.4	6417 [2796]	NA
Toosizadeh et al. ^30^, 2015	15	71.2 [6.3]	5.9 [5.3]	2.8	4099 [2673]	0.66 [0.11]
Weiss et al. ^47^, 2014	40 (fallers)	66.5 [8.2]	6.1 [4.0]	2.9	3131 [3097]	NA
	67 (non-fallers)	64.0 [9.8]	5.2 [3.1]	2.4	3553 [3257]	NA
Nakae et al. ^48^, 2014	10	NA	12.6 [5.6]	NA	NA	0.83
Wallén et al. ^49^, 2014	66	73.1 [5.8]	NA	2.5	4730 [3210]	NA
Lord et al. ^50^, 2013	89	67.3 [9.9]	NA	2.0	5452 [2501]	NA
Cavanaugh et al. ^51^, 2012	33	67.1 [8.8]	4.4 [4.2]	2.4	10,261 [4333]	NA
Nakae et al. ^52^, 2011	9	66.4 [5.3]	9.2 [2.2]	NA	NA	0.99
Ford et al. ^53^, 2010	12	NA	NA	2.0	8996	NA
Sue Lord et al. ^29^, 2008	12	70.5 [3.3]	8.0 [3.0]	2.9	NA	0.83 [0.16]
Skidmore et al. ^54^, 2008	24	70.0 [9.0]	7.5 [3.8]	2.7	3981 [1448]	NA
Xanthopoulos et al. ^55^, 2008	16	71.0 [11.0]	7.0 [4.2]	NA	4378 [2057]	NA
Busse et al. ^56^, 2004	10	67.1 [8.2]	NA	NA	3818	0.99 [0.16]

All estimated measures reported in this table are mean [standard deviation] values unless otherwise noted. NA not available. Studies were selected from a PubMed search using “Gait activity”, “Gait speed”, and “Parkinson’s disease” and included studies where wearable sensors were worn outside a clinical setting in the real world.

Importantly, our study analyzes tremors at home and quantifies the duration, frequency, and amplitude of tremors experienced by individuals with PD over the course of their daily lives outside the clinic. Tremor amplitude was dependent on the activity with the highest tremor amplitudes occurring in sitting intervals and lowest during the lying down (and likely asleep) intervals. The latter finding is consistent with prior reports indicating that the rest tremor in PD abates during sleep^32^. To our knowledge, this is the first time tremor has been objectively measured in relation to activity over significantly longer durations that includes a full diurnal cycle. Data from our wearable sensor-based study also allows us to assess the proportion of time over which individuals with PD experience tremors within the course of their normal lives. Participants with PD in our study experienced tremors in their most-affected hands for a median [range] 1.6 [0.4–5.9] h/day. Notably, we also found that individuals with an MDS-UPDRS 3.17a or 3.17b score of zero (considered a “less-affected hand” in our study), had a tremor in this hand for median [range] 0.5 [0.3–2.3] h/day. This finding is further evidence that episodic in-clinic assessments and clinical scales may miss symptoms that are variable. A clinical-epidemiological study^33^, conducted in 2016, recruited 100 individuals with PD who underwent standard clinical assessments, which included a neurological examination and a standard questionnaire. In the questionnaire, the participants were asked to mention the total number of hours with tremors on a typical day. Based on the questionnaire response data collected, the study reported a median of 3 h with tremor which was higher than the median of 1.6 h reported in our study. Part of the difference could be explained by the fact that while the questionnaire responses only indicated the aggregate durations of tremors, our analysis focused on sensors placed on the forearm which likely missed tremors in other regions of the body, such as the thumb, chin, and legs. We also eliminated walking portions during analysis which could have excluded re-emergent tremor. A wearable sensor-based study^34^ performed 24-h monitoring of 25 participants with PD (average Hoehn and Yahr stage of 3.5 and MDS-UPDRS motor score of 46.0) in a rehabilitation center. This study reported that the tremor activity was high in the morning and reduced in the night, a finding similar to what we have reported. Also, the study reports that the average tremor proportion of 18.5% and 10% in the sitting and standing/walking intervals in comparison with 14.8% and 9.7% during the sitting and standing intervals, respectively, in our study. The difference in the tremor proportion in the sitting intervals can be attributed to the fact that the individuals in the mentioned study had more advanced PD in comparison with the individuals in our study. Another wearable sensor study^21^ analyzed tremors of 13 PD participants during the OFF and ON medication over a 2-h duration during which the participants performed six activities of daily living. The study showed a median tremor proportion of ≈35% during OFF medication which was higher than ≈28% found during the in-clinic OFF medication (that comprised of UPDRS assessments for about 0.5 h) for the most-affected hand in our study. Finally, and importantly, our tremor analysis revealed a difference between individuals classified as having tremor-dominant PD vs. those with postural instability/gait difficulty PD. Although these findings should be replicated in larger studies, it demonstrates the potential of wearable devices for use as objective measures and/or disease classification in PD.

While the study provides new insights, it is not without limitations. The study had a relatively small sample with generally mild disease. The stage of PD can affect the analyses we performed and we plan to stratify by disease stage in larger studies. While the wearable-sensor-based motor analysis for in-clinic durations could be validated by cross-checking against the video recorded in the clinic, for the real-world data outside of the clinic, the participant activity diaries varied in detail and could not provide validation comparable to the video. Some participants provided detailed activity logs which were helpful; one such instance identified high tremor amplitude durations during the waking lying down intervals for a participant with PD (described in the Results section). The rhythmicity index, which measures the amplitude of the rhythmic motion in hands, allowed us to identify intervals with high amplitude rhythmic motion of the hands. Although by using an appropriate threshold, we are able to exclude most deliberate movements when identifying intervals with tremors, one current limitation is that we cannot eliminate deliberate rhythmic movements that have a frequency typical of tremors. In-clinic duration activities such as finger tapping and pronation/supination and real-world activities such as brushing teeth and scrubbing dishes result in high rhythmicity index, thus contributing to the tremor proportion. However, such deliberate rhythmic motions occur infrequently and for rather short durations as evidenced by a small median tremor proportion of 0.4 h/day for control participants. Our specific sensor placements posed problems for the analysis of leg tremor analogous to the hand tremor analysis presented in this paper. Specifically, from checking the in-clinic videos for the participants, we found that several of the participants rested their hands on the leg sensor when sitting due to which the leg sensor also recorded the hand tremor. Future studies should explore alternative leg sensor placement to avoid this cross-contamination.

Our study, which started as a single point observational study allowed participants that were willing to participate in longitudinal observations at six, nine, and twelve months from the initial observation. Although accelerometer data from the wearable sensors were recorded for these additional visits, the meaningful longitudinal analysis could not be conducted because the participants had several changes in medications and dosage over the course of the study and the number of participants was too small to attempt analyses of smaller groups in which these parameters were constant over the longitudinal duration.

In this study, wearable sensors added to evidence that gait activity is reduced in individuals with PD in their natural environment. In addition, an innovative algorithm combined with multiple sensors was able to quantify the amplitude of rest tremor, determine its onset and termination, its relationship to physical states, and its total duration in the real world. These new insights advance our understanding of the motor features of PD and could in the future provide valuable information to improve care and enhance the evaluation of therapies. Larger and longer-duration studies are required to replicate these findings and to evaluate how they change over time.

## Methods

### Study overview and design

We conducted an observational study in individuals with PD and in controls without a movement disorder using accelerometers packaged in BioStampRC^®^ wearable sensors developed by MC10 Inc. (Lexington, MA, USA). The University of Rochester’s institutional review board approved the procedures used in the study, and there was full compliance with human experimentation guidelines. We recruited individuals with PD from clinics, study interest registries, and regional support groups. Control participants were comprised of unaffected spouses, family members, friends, and community members. Participants with PD had at least two of the four cardinal features (rest tremor, bradykinesia, cogwheel rigidity, and difficulty with gait or balance) on the exam and no better alternative explanation for the condition as determined by the investigators. All participants provided written informed consent before study participation.

**Fig. 2:**
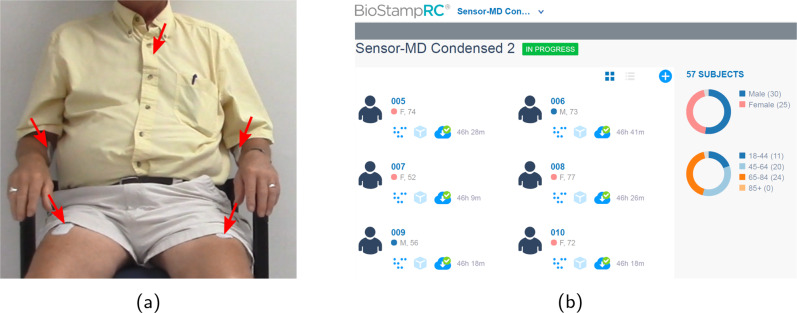
Sensor placement and cloud-based web portal for accessing data. **a** A study participant wearing the sensors at five different locations on the trunk and each limb, and **b** web-portal for accessing the recorded sensor data over the duration of sensor wear. As part of the written consent for participation in the study, the participant shown in (**a**) provided permission for their image to be used.

In the clinic, participants underwent the Montreal Cognitive Assessment^35^ and signed a video waiver form before providing basic demographic data and medical history. Participants were outfitted with five self-adhesive wearable accelerometer sensors with one on each anterior thigh, one on each anterior forearm, and one on the trunk as shown in Fig. 2a. Participants were video-recorded undergoing standard clinic assessments including the Movement Disorder Society—Unified Parkinson Disease Rating Scale (MDS-UPDRS) Part III^36^, Timed Up-and-Go^37^, and Ten-Meter Walk test^38^. Three physicians experienced in PD (JA, CT, SS) and certified by the MDS-UPDRS motor score online certification^39^ performed all motor assessments.

After the in-clinic (approximately 1 h) assessments, participants wore the sensors in the real world (out of clinic) for an additional continuous interval totaling approximately 44 h (sensors were worn during sleep). Participants were asked to complete an activity log to supplement the sensor data for the non-clinic durations. The activity log included information about daily activities along with information on the participant’s PD medication schedule, if applicable. An example of an activity log is shown in Supplementary Table 1. At the end of the 2-day real-world monitoring period, the sensors and activity logs were mailed back to the research team, and the data were extracted via Bluetooth and uploaded to the MC10 cloud storage through Wi-Fi. The raw sensor data stored in the MC10 cloud were accessed and downloaded via the MC10 Web Portal, shown in Fig. 2b, and used for analyses.

### Wearable sensor data collection

Tri-axial accelerometer data were collected from the sensors at a sampling rate of 31.25 Hz. The activity states were determined based on posture information obtained using the data from the trunk sensor and thigh sensors. The gait and the tremor analyses were performed in the walking and non-walking states, respectively, identified during the activity analysis. Due to the rhythmic nature of the walking activity, the tremor analysis excluded the walking intervals.

### Activity analysis

Each sensor observation period was partitioned into non-overlapping 2 s windows and for each 2 s window, activity analysis was performed using a previously described technique^8^. For each 2-s interval, a posture was determined based on the combination of the dominant axis (*x*, *y*, or *z*) for the trunk and thigh sensors. The postures were categorized as lying down, sitting, and standing/walking. Walking durations were further distinguished from standing by identifying the quasi-periodic acceleration patterns associated with walking by using the normalized auto-correlation analysis of the trunk sensor data as previously described^40^.

### Gait analysis

Using the accelerometer data obtained from trunk and thigh sensors during each 2-s walking interval, we estimated step count, step duration, gait speed, and coordination between the legs for each participant using previously developed techniques^40^. Periodic steps while walking result in strong auto-correlation peaks at lags corresponding to the step duration. Hence normalized auto-correlation of trunk sensor data was used to estimate step count and step duration. Unlike methods that count steps by matching against templates developed for controls, the auto-correlation is computed from data for a single participant, and the methodology, therefore, has the advantage that it adapts to individual impairments in gait, although it may miss counting individual isolated steps. To estimate coordination between legs while walking, we computed normalized cross-correlation between left and right leg sensor data. The strength of the cross-correlation peaks at a one-step lag characterize how well sensor data from one (left) leg predicts the sensor data for the other (right) leg, serving as a proxy for coordination. Step length was estimated using an empirical method^41^. Step length was divided by step duration to estimate the gait speed.

### Tremor analysis

The tremor analysis aimed to quantify PD tremor, which is rhythmic in nature, has a typical frequency and is more prevalent in the hands^14^. Since different hands exhibited different ranges of tremor amplitude and frequency, we analyzed “most-affected” and “less-affected” hands separately. A hand was considered “most-affected” if the MDS-UPDRS maximal at-rest tremor score (MDS-UPDRS 3.17a–3.17b) for the hand ranged from 1 to 4 and “less-affected” hands were identified by an MDS-UPDRS maximal at-rest tremor score of 0. For the PD cohort, there were 16/34 (47%) most-affected hands and 18/24 (53%) less-affected hands. The hands of all control participants had an MDS-UPDRS maximal at-rest tremor score of 0 and the right hand was analyzed. Using the accelerometer data obtained from the forearm sensors during each 2-s non-walking interval, we computed a rhythmicity index and a peak frequency (over a frequency range of 3–10 Hz), which represented the amplitude and frequency of rhythmic motion of the hands, respectively. Based on the in-clinic video assessment, a threshold value was then determined to separate intervals with rhythmic movements from those without. The (estimated) tremor proportion was quantified as the fraction of two-second intervals for which the rhythmicity index exceeded the threshold. The following section provides a detailed description of the algorithm used for the tremor analysis.

### Algorithm for computing rhythmicity index, peak frequency, and tremor proportion

To compute rhythmicity index and peak frequency, we first performed a moving mean subtraction on the recorded 3D accelerometer data to remove the effect of gravity. Next, to obtain the direction of dominant hand motion, we applied principal component analysis^42^ to the mean subtracted 3D accelerometer data and chose the first principal component. The first principal component was then analyzed and visualized in the frequency domain using spectrogram analysis^43^. Since our focus was on estimating the amplitude and frequency of rhythmic motion of the hands, parameters of spectrogram analysis were chosen to provide a high-frequency resolution. From the spectrogram we calculated the magnitude short term Fourier transform (STFT) and integrated it along the time axis to obtain time-integrated magnitude STFT (TIM-STFT). The presence of rhythmic motion showed a clear signature in the TIM-STFT: around the fundamental frequency and its harmonics, the TIM-STFT clearly exhibited sharp peaks that tapered down to relatively low values for neighboring frequency regions in either side. These peaks were absent in the absence of rhythmic motion and a higher peak to neighborhood amplitude ratio represented a higher amplitude of rhythmic motion in the hands. For quantitative evaluation, a peak frequency and rhythmicity index were therefore computed as follows. First, the frequency corresponding to the peak in the TIM-STFT occurring in the 3–10 Hz tremor frequency range was identified as the peak frequency and an approximate bandwidth of 1 Hz around the peak frequency was identified as the peak region. Frequency bands with 1 Hz bandwidth located 2 Hz away from the peak on either side were identified as the neighborhood region. The rhythmicity index was then computed as the ratio of the sums of the TIM-STFT in the afore-mentioned peak and neighborhood regions.

Based on the in-clinic video assessment, as described in the following, a threshold value was then determined to separate intervals with rhythmic movements from those without. From the participant group, two PD and two control participants were chosen. The in-clinic sensor data for these participants were synchronized with the corresponding in-clinic videos. The rhythmicity index was computed for 2-s intervals, and the synchronized videos for the corresponding durations were manually examined to identify whether or not these included rhythmic movements (either due to tremor or voluntary), and a threshold was determined such that the computed rhythmicity index for intervals with typical non-rhythmic deliberate movements was below the threshold and for intervals with rhythmic movements (deliberate or not) was above the threshold. Note that a high rhythmicity index can be a result of deliberate (e.g., in-clinic assessment activities such as finger tapping, pronation/supination of hands) or non-deliberate (e.g., PD tremor) rhythmic motion of the hands. Among PD participants with tremors, however, the deliberate rhythmic motion intervals occupy a much smaller fraction of time than non-deliberate rhythmic motion intervals. To quantify the relative amount of time with high amplitude rhythmic motion, we computed tremor proportion as the fraction of 2-s intervals for which the rhythmicity index exceeded the threshold.

### Statistical analysis

Due to the small sample size, we chose to perform non-parametric tests to analyze differences in activity, gait, and tremor between PD and control groups. To assess differences in activity (proportion of time spent lying, sitting, standing, and walking) and gait parameters (step count, step duration, gait speed, and co-ordination between legs) between PD and control groups and to assess pairwise differences in tremor proportion between the most-affected and less-affected hands of PD and the right hand of control participant groups, we analyzed the data using the Wilcoxon rank-sum test^44^. The Spearman correlation coefficient^44^ was used to assess the relationship between the proportion of the observed period with tremor and clinician-rated MDS-UPDRS maximal at-rest tremor score. All the hypothesis tests were one-sided and a significance level of *P* = 0.05 was used. Median and range/interquartile range were reported as summary statistics. Statistical analysis was performed using MATLAB^®^ (version 2019b, MathWorks, Natick, MA).

### Reporting summary

Further information on research design is available in the Nature Research Reporting Summary linked to this article.

## Supplementary information

Supplementary Information

Reporting Summary

## Data Availability

The sensor accelerometry and MDS-UPDRS assessment-task annotation data for each participant, and demographic and clinical assessment data for all participants are available at IEEE DataPort with identifier “10.21227/g2g8-1503”^45^.

## Code availability

The code is being considered for licensing by the University Technology Transfer office and is therefore not publicly available. We expect that upon licensing and deployment of the code, other researchers using these sensor systems will be able to access the analysis via a cloud-based service.
