# Supplementary material for: A real-world study of wearable sensors in Parkinson’s disease
Source: NPJ Parkinsons Dis. 2021 Nov 29;7:106. doi: 10.1038/s41531-021-00248-w (PMC8629990; doi:10.1038/s41531-021-00248-w)
Supplement: Supplementary file 1 — Supplementary Information [file 41531_2021_248_MOESM1_ESM.pdf]

Supplementary Online Content

A real-world study of wearable sensors in Parkinson's disease

+*Jamie L. Adams, MD[b,d]; +Karthik Dinesh, MS[a] ; Christopher W Snyder, MS[e]; Mulin Xiong, BA[c]; Christopher G. Tarolli, MD[b,d]; Saloni Sharma, MD[b]; E. Ray Dorsey, MD[b,d]; Gaurav Sharma, PhD[a]

[a]Department of Electrical and Computer Engineering, University of Rochester, Rochester, NY, USA
[b]Center for Health + Technology, University of Rochester Medical Center, Rochester, NY, USA
[c]Michigan State University College of Human Medicine, East Lansing, MI, USA
[d]Department of Neurology, University of Rochester Medical Center, Rochester, NY, USA
[e]Rochester General Hospital, Microbiology, Rochester, NY, USA
+ indicates equal contribution

Supplementary Figure 1. Comparison of tremor proportion distribution over sitting and standing duration for most-affected and less-affected hand of participants with PD and right hand of control participants

Supplementary Note 1. Multiple comparison analysis for hand tremor

Supplementary Figure 2. Rhythmicity index clock visualizations for most-affected hand of participants with PD

Supplementary Figure 3. Rhythmicity index clock visualizations for less-affected hand of participants with PD

Supplementary Figure 4. Rhythmicity index clock visualizations for control participants

Supplementary Figure 5. Scatter plots showing rhythmicity index and peak frequency values for the most-affected hand and less-affected hands of participants with PD and right hand of control participants over in-clinic duration

Supplementary Figure 6. Heat maps showing rhythmicity index and peak frequency values for the most-affected and less-affected hand of participants with PD and right hand of control participants over real-world (out of clinic) durations

Supplementary Table 1. An example of activity log used in the study

Supplementary Figure 1: Comparison of tremor proportion distribution over sitting and standing duration for most-affected and less-affected hand of participants with PD and right hand of control participants

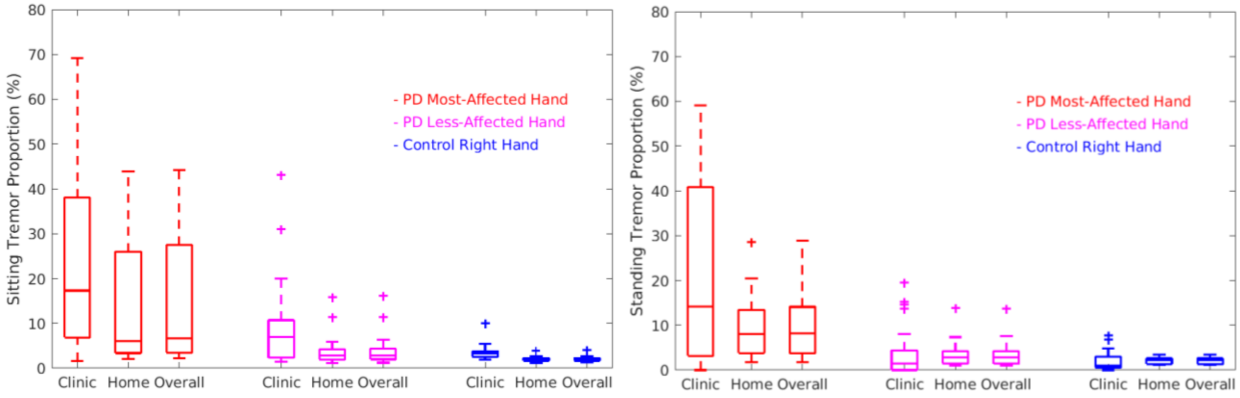

(a) (b)

Supplementary Figure 1: Box plots showing the tremor proportion distribution over (a) sitting intervals and (b) standing intervals during clinic, real-world, and overall duration of the sensor wear, for the most-affected and less-affected hand of PD participants and right hand of the control participants. The median tremor proportion over sitting and standing durations for the most-affected hand of PD participants was higher than that of less-affected hand of the PD and right hand of the control participants.

Supplementary Note 1: Multiple comparison analysis for hand tremor

We performed a Kruskal-Wallis test and found that the median hand tremor proportion between the groups (most-affected and less-affected hand of the PD participants and right hand of the unaffected control participants) were significantly different ($P < 0.001$). Post hoc multiple comparison tests revealed that hand tremor proportions between each pair of groups were also significantly different (most-affected hand of PD and less-affected hand of PD; $P = 0.05$, most-affected hand of PD and right hand of unaffected control; $P < 0.001$, less-affected hand of PD and right hand of unaffected control; $P = 0.04$).

Supplementary Figure 2: Rhythmicity index clock visualizations for most-affected hand of participants with PD

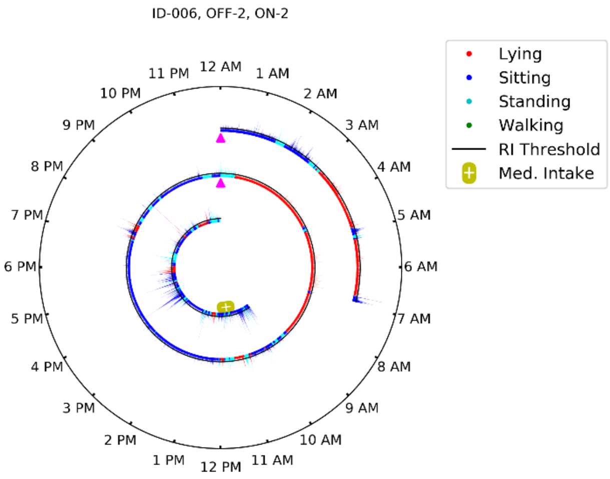

(a)

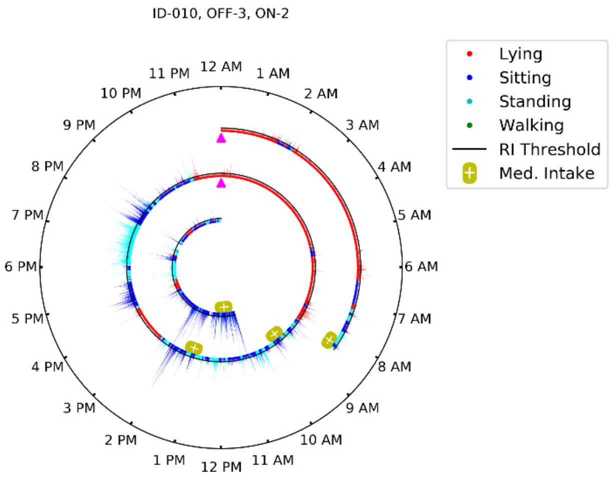

(b)

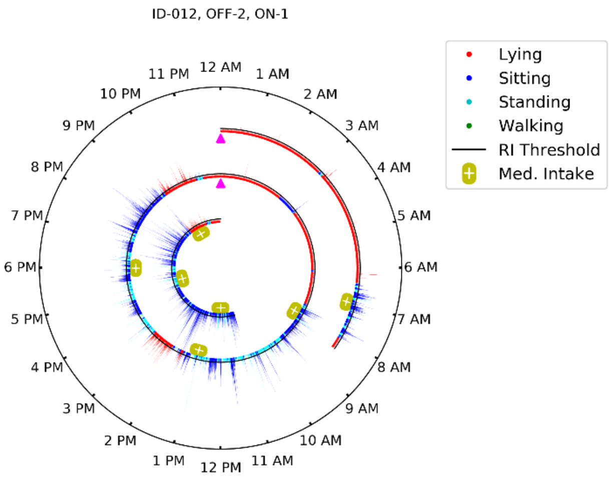

(c)

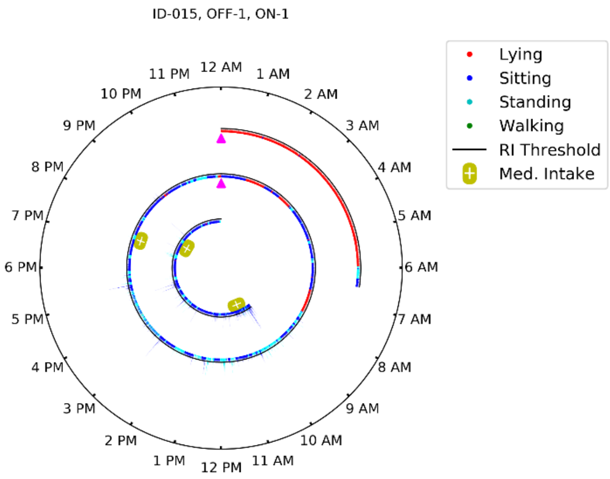

(d)

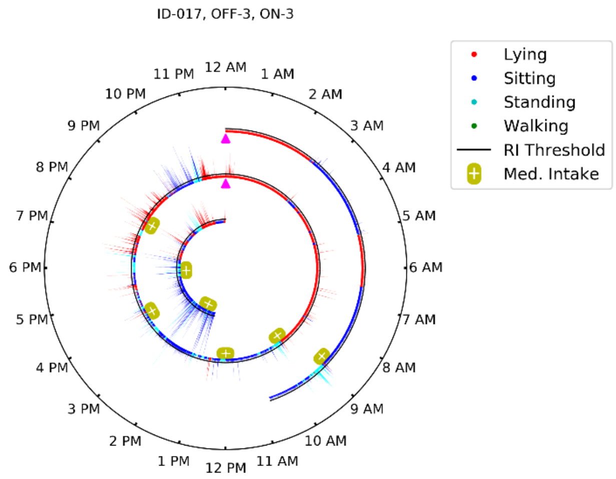
ID-017, OFF-3, ON-3
12 AM
1 AM
2 AM
3 AM
4 AM
5 AM
6 AM
7 AM
8 AM
9 AM
10 AM
11 AM
12 PM
1 PM
2 PM
3 PM
4 PM
5 PM
6 PM
7 PM
8 PM
9 PM
10 PM
11 PM
Lying
Sitting
Standing
Walking
RI Threshold
Med. Intake

(e)

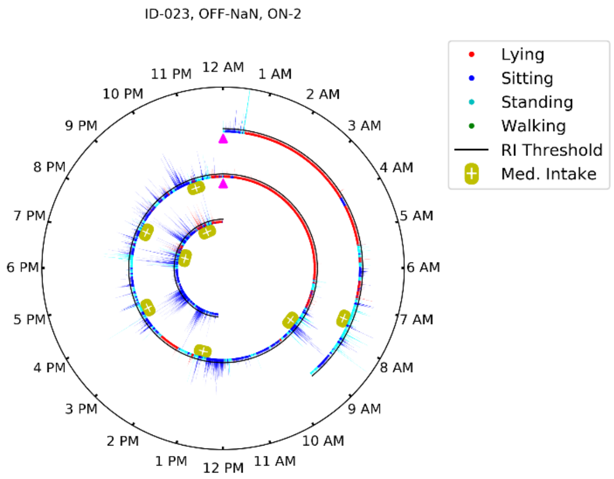
ID-023, OFF-NaN, ON-2
12 AM
1 AM
2 AM
3 AM
4 AM
5 AM
6 AM
7 AM
8 AM
9 AM
10 AM
11 AM
12 PM
1 PM
2 PM
3 PM
4 PM
5 PM
6 PM
7 PM
8 PM
9 PM
10 PM
11 PM
Lying
Sitting
Standing
Walking
RI Threshold
Med. Intake

(f)

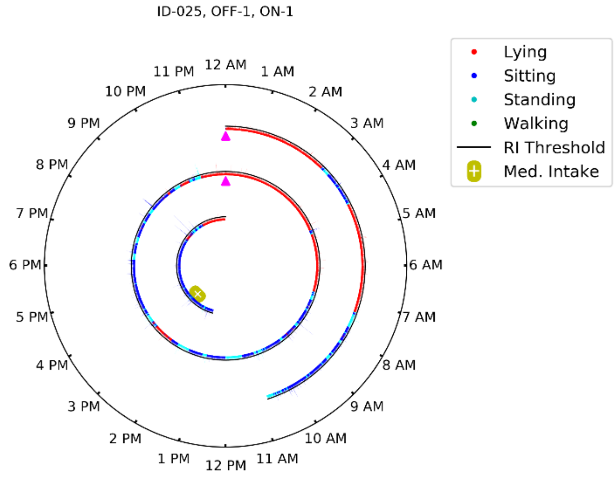
ID-025, OFF-1, ON-1
12 AM
1 AM
2 AM
3 AM
4 AM
5 AM
6 AM
7 AM
8 AM
9 AM
10 AM
11 AM
12 PM
1 PM
2 PM
3 PM
4 PM
5 PM
6 PM
7 PM
8 PM
9 PM
10 PM
11 PM
Lying
Sitting
Standing
Walking
RI Threshold
Med. Intake

(g)

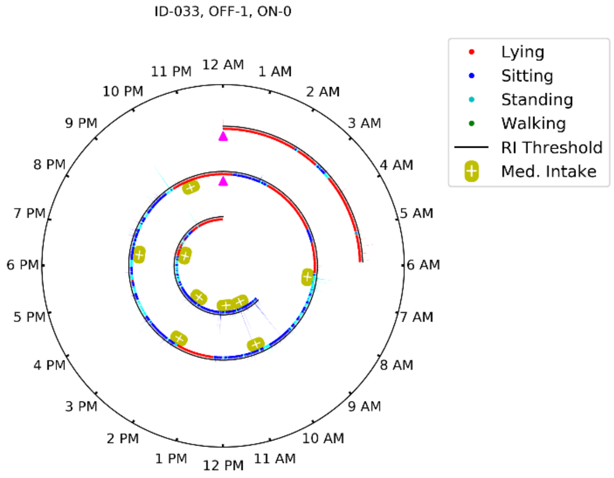
ID-033, OFF-1, ON-0
12 AM
1 AM
2 AM
3 AM
4 AM
5 AM
6 AM
7 AM
8 AM
9 AM
10 AM
11 AM
12 PM
1 PM
2 PM
3 PM
4 PM
5 PM
6 PM
7 PM
8 PM
9 PM
10 PM
11 PM
Lying
Sitting
Standing
Walking
RI Threshold
Med. Intake

(h)

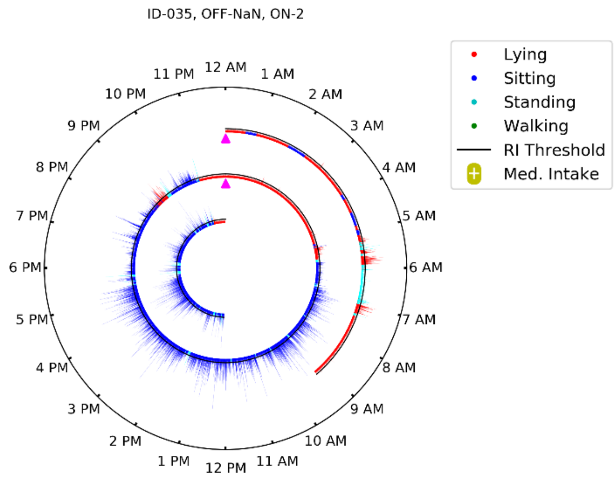
ID-035, OFF-NaN, ON-2
12 AM
1 AM
2 AM
3 AM
4 AM
5 AM
6 AM
7 AM
8 AM
9 AM
10 AM
11 AM
12 PM
1 PM
2 PM
3 PM
4 PM
5 PM
6 PM
7 PM
8 PM
9 PM
10 PM
11 PM
Lying
Sitting
Standing
Walking
RI Threshold
Med. Intake

(i)

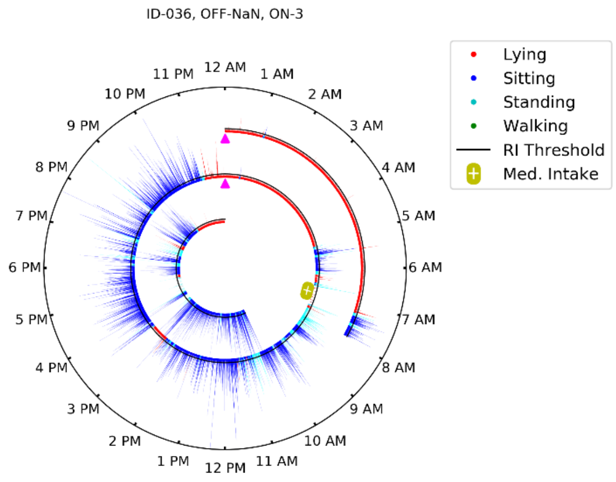
ID-036, OFF-NaN, ON-3
12 AM
1 AM
2 AM
3 AM
4 AM
5 AM
6 AM
7 AM
8 AM
9 AM
10 AM
11 AM
12 PM
1 PM
2 PM
3 PM
4 PM
5 PM
6 PM
7 PM
8 PM
9 PM
10 PM
11 PM
Lying
Sitting
Standing
Walking
RI Threshold
Med. Intake

(j)

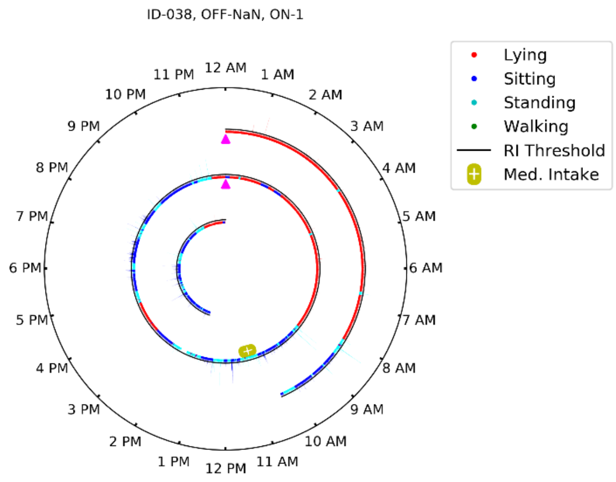
ID-038, OFF-NaN, ON-1
12 AM
1 AM
2 AM
3 AM
4 AM
5 AM
6 AM
7 AM
8 AM
9 AM
10 AM
11 AM
12 PM
1 PM
2 PM
3 PM
4 PM
5 PM
6 PM
7 PM
8 PM
9 PM
10 PM
11 PM
Lying
Sitting
Standing
Walking
RI Threshold
Med. Intake

(k)

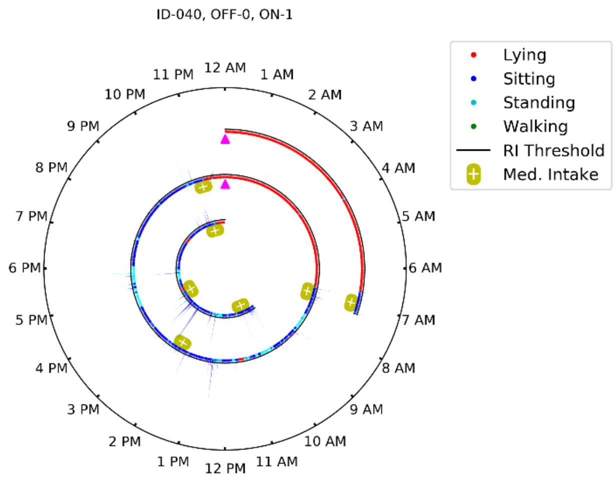
ID-040, OFF-0, ON-1
12 AM
1 AM
2 AM
3 AM
4 AM
5 AM
6 AM
7 AM
8 AM
9 AM
10 AM
11 AM
12 PM
1 PM
2 PM
3 PM
4 PM
5 PM
6 PM
7 PM
8 PM
9 PM
10 PM
11 PM
Lying
Sitting
Standing
Walking
RI Threshold
Med. Intake

(l)

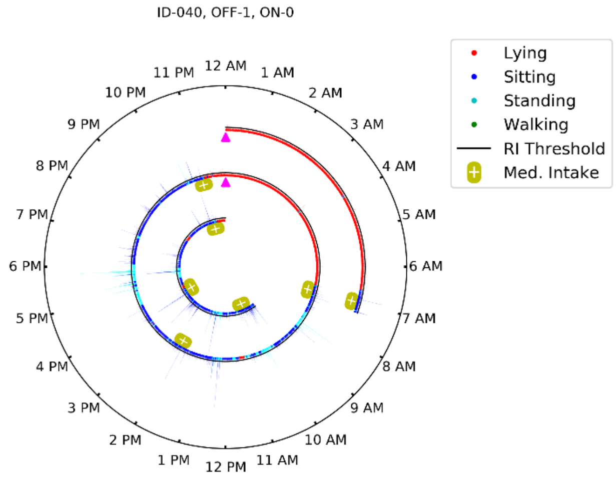

(m)

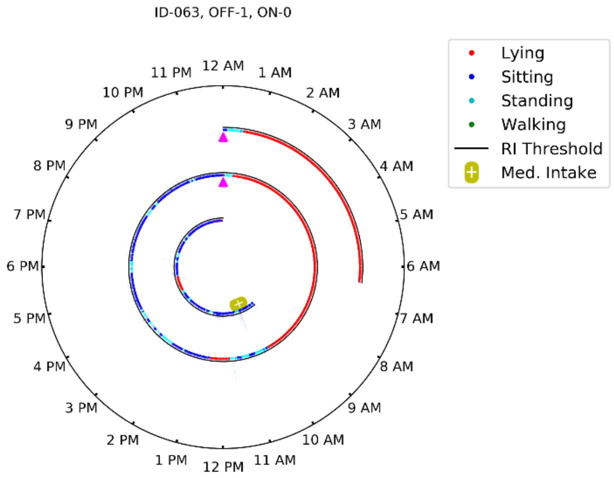

(n)

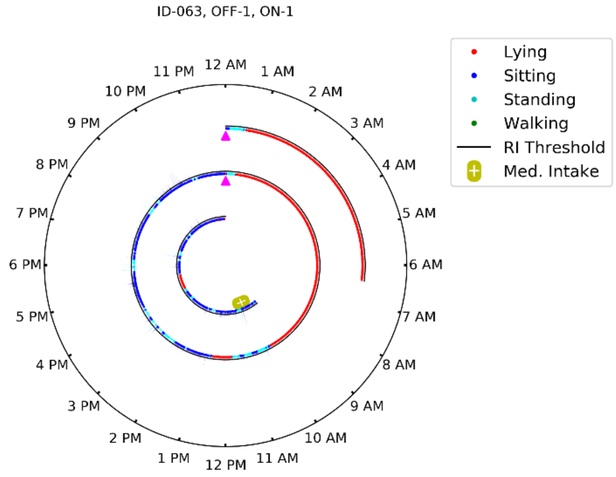

(o)

Supplementary Figure 2: Rhythmicity index profiles over full duration of the sensor wear augmented with the activity clock and illustrated for the most-affected hand of the participants with PD. Text annotations on the graph indicate the participant ID and the off/on MDS-UPDRS maximal at-rest tremor amplitude scores. OFF – NaN indicates that the participant performed only the on medication MDS-UPDRS assessments. Each color-coded bar on the polar plot jointly represents the rhythmicity index and activity state (lying, sitting, standing, or walking) over a 2-second interval, with the color identifying the activity state and the height of the bar indicating the rhythmicity index. The black circle above each radius represents the rhythmicity index threshold, which is set to a value of 3.3. The yellow capsule-shaped markers below each radius represent the medication intake times.

Supplementary Figure 3: Rhythmicity index clock visualizations for less-affected hand of participants with PD

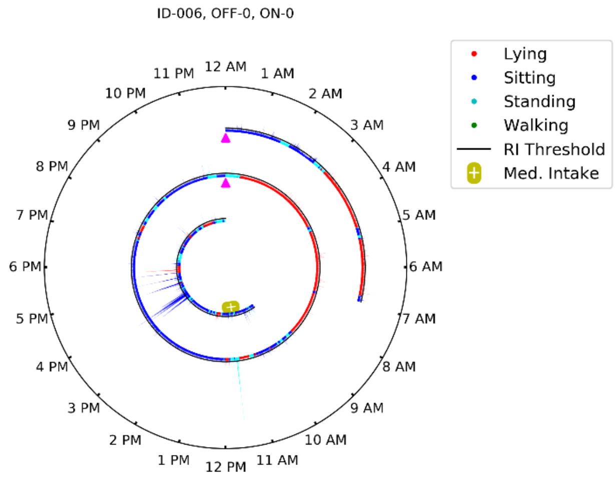

(a)

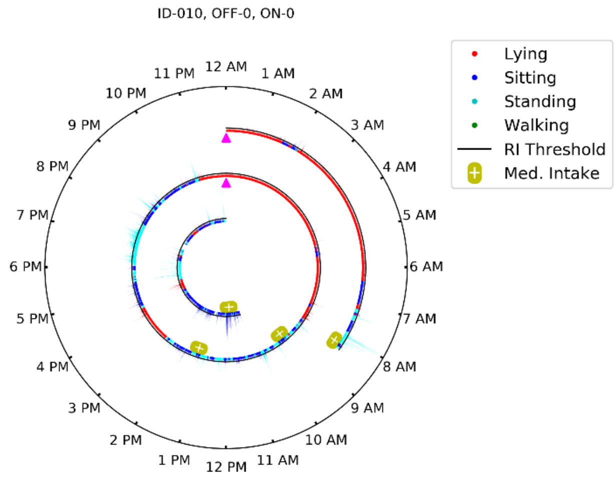

(b)

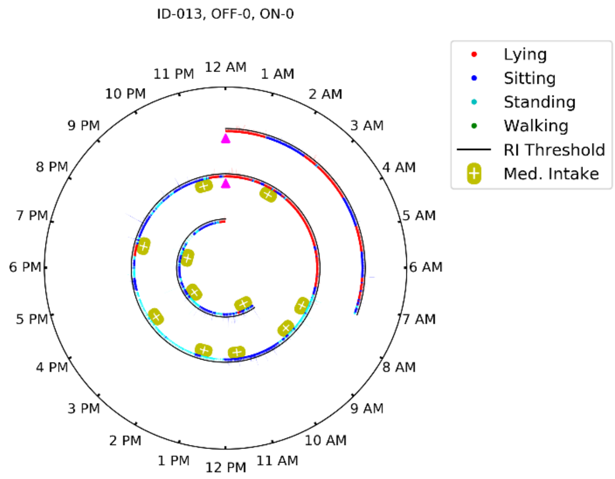

(c)

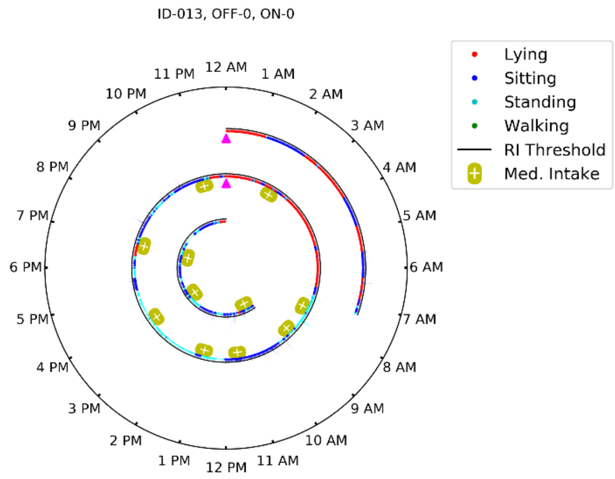

(d)

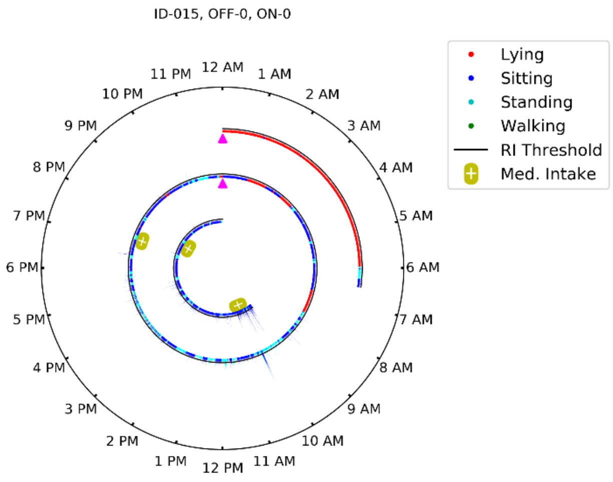
ID-015, OFF-0, ON-0
12 AM
1 AM
2 AM
3 AM
4 AM
5 AM
6 AM
7 AM
8 AM
9 AM
10 AM
11 AM
12 PM
1 PM
2 PM
3 PM
4 PM
5 PM
6 PM
7 PM
8 PM
9 PM
10 PM
11 PM
Lying
Sitting
Standing
Walking
RI Threshold
Med. Intake

(e)

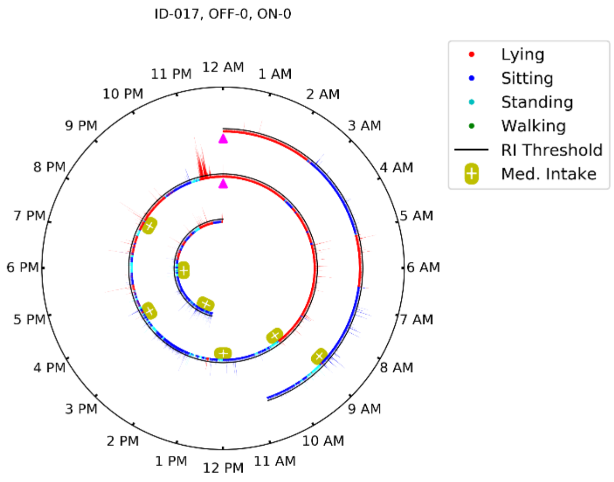
ID-017, OFF-0, ON-0
12 AM
1 AM
2 AM
3 AM
4 AM
5 AM
6 AM
7 AM
8 AM
9 AM
10 AM
11 AM
12 PM
1 PM
2 PM
3 PM
4 PM
5 PM
6 PM
7 PM
8 PM
9 PM
10 PM
11 PM
Lying
Sitting
Standing
Walking
RI Threshold
Med. Intake

(f)

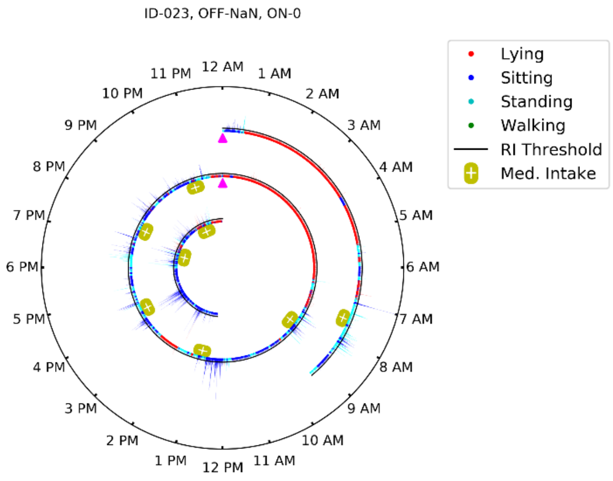
ID-023, OFF-NaN, ON-0
12 AM
1 AM
2 AM
3 AM
4 AM
5 AM
6 AM
7 AM
8 AM
9 AM
10 AM
11 AM
12 PM
1 PM
2 PM
3 PM
4 PM
5 PM
6 PM
7 PM
8 PM
9 PM
10 PM
11 PM
Lying
Sitting
Standing
Walking
RI Threshold
Med. Intake

(g)

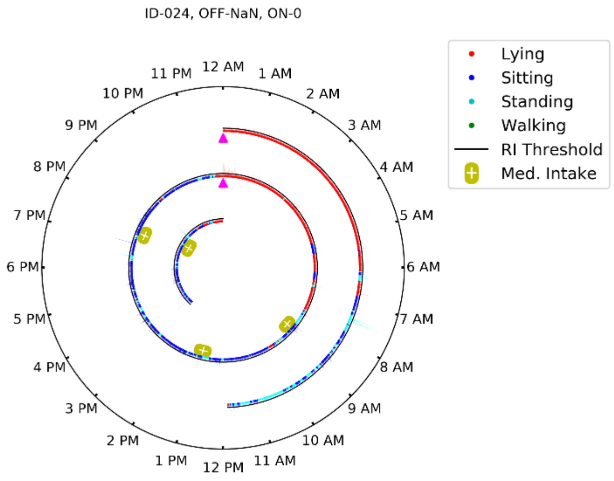
ID-024, OFF-NaN, ON-0
12 AM
1 AM
2 AM
3 AM
4 AM
5 AM
6 AM
7 AM
8 AM
9 AM
10 AM
11 AM
12 PM
1 PM
2 PM
3 PM
4 PM
5 PM
6 PM
7 PM
8 PM
9 PM
10 PM
11 PM
Lying
Sitting
Standing
Walking
RI Threshold
Med. Intake

(h)

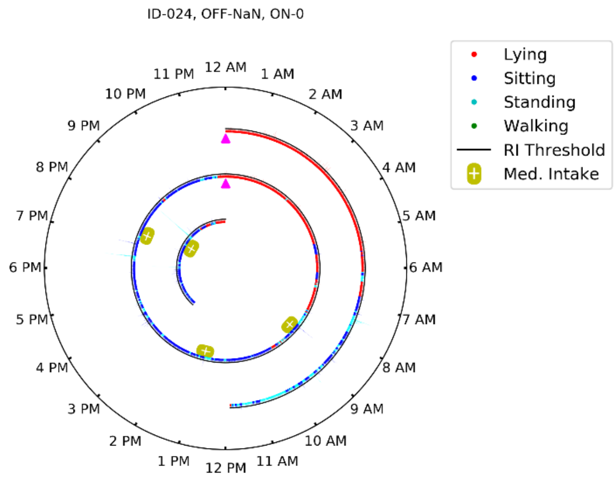
ID-024, OFF-NaN, ON-0
12 AM
1 AM
2 AM
3 AM
4 AM
5 AM
6 AM
7 AM
8 AM
9 AM
10 AM
11 AM
12 PM
1 PM
2 PM
3 PM
4 PM
5 PM
6 PM
7 PM
8 PM
9 PM
10 PM
11 PM
Lying
Sitting
Standing
Walking
RI Threshold
Med. Intake

(i)

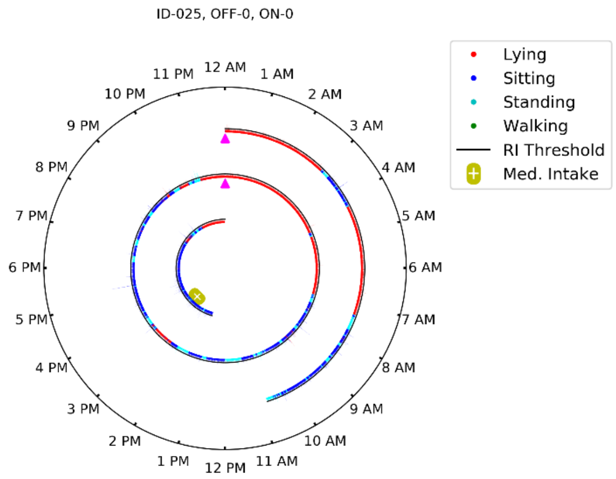
ID-025, OFF-0, ON-0
12 AM
1 AM
2 AM
3 AM
4 AM
5 AM
6 AM
7 AM
8 AM
9 AM
10 AM
11 AM
12 PM
1 PM
2 PM
3 PM
4 PM
5 PM
6 PM
7 PM
8 PM
9 PM
10 PM
11 PM
Lying
Sitting
Standing
Walking
RI Threshold
Med. Intake

(j)

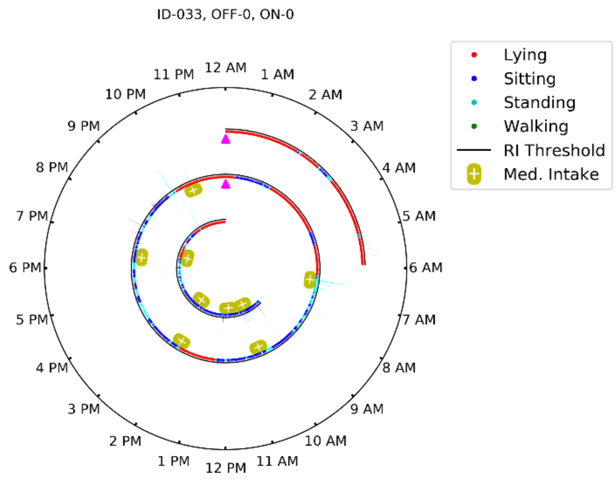
ID-033, OFF-0, ON-0
12 AM
1 AM
2 AM
3 AM
4 AM
5 AM
6 AM
7 AM
8 AM
9 AM
10 AM
11 AM
12 PM
1 PM
2 PM
3 PM
4 PM
5 PM
6 PM
7 PM
8 PM
9 PM
10 PM
11 PM
Lying
Sitting
Standing
Walking
RI Threshold
Med. Intake

(k)

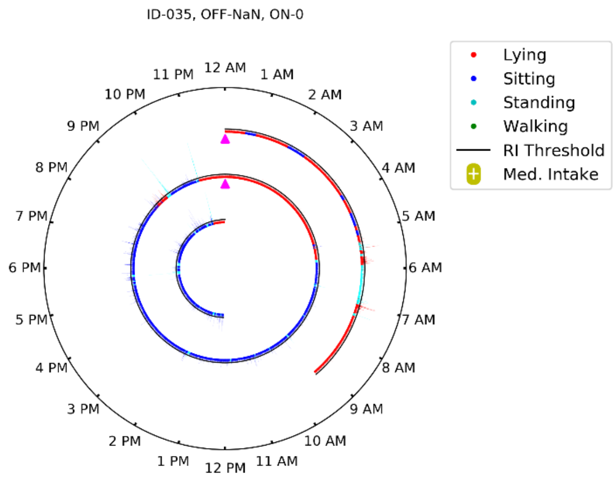
ID-035, OFF-NaN, ON-0
12 AM
1 AM
2 AM
3 AM
4 AM
5 AM
6 AM
7 AM
8 AM
9 AM
10 AM
11 AM
12 PM
1 PM
2 PM
3 PM
4 PM
5 PM
6 PM
7 PM
8 PM
9 PM
10 PM
11 PM
Lying
Sitting
Standing
Walking
RI Threshold
Med. Intake

(l)

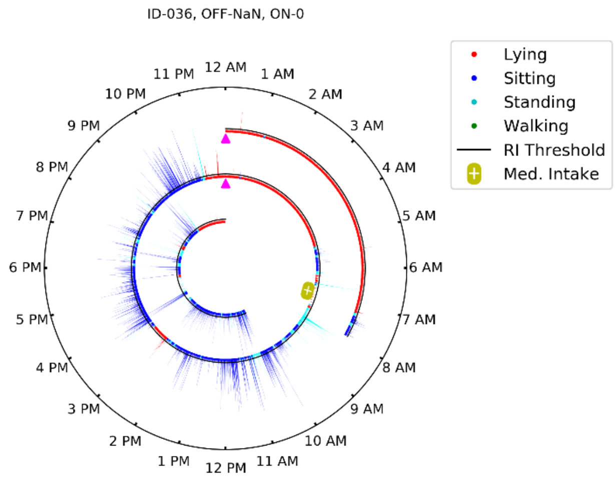
ID-036, OFF-NaN, ON-0
12 AM
1 AM
2 AM
3 AM
4 AM
5 AM
6 AM
7 AM
8 AM
9 AM
10 AM
11 AM
12 PM
1 PM
2 PM
3 PM
4 PM
5 PM
6 PM
7 PM
8 PM
9 PM
10 PM
11 PM
Lying
Sitting
Standing
Walking
RI Threshold
Med. Intake

(m)

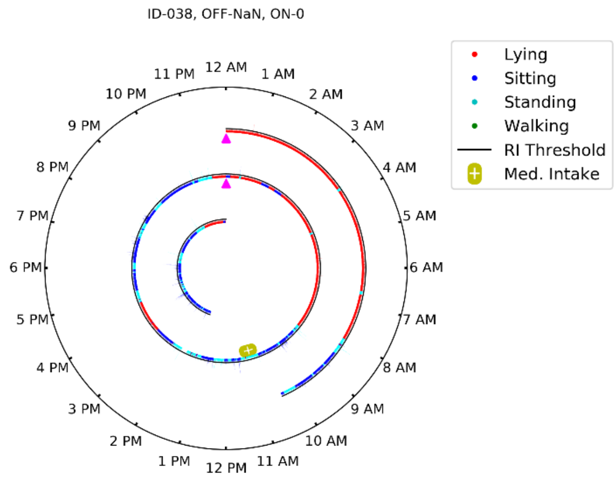
ID-038, OFF-NaN, ON-0
12 AM
1 AM
2 AM
3 AM
4 AM
5 AM
6 AM
7 AM
8 AM
9 AM
10 AM
11 AM
12 PM
1 PM
2 PM
3 PM
4 PM
5 PM
6 PM
7 PM
8 PM
9 PM
10 PM
11 PM
Lying
Sitting
Standing
Walking
RI Threshold
Med. Intake

(n)

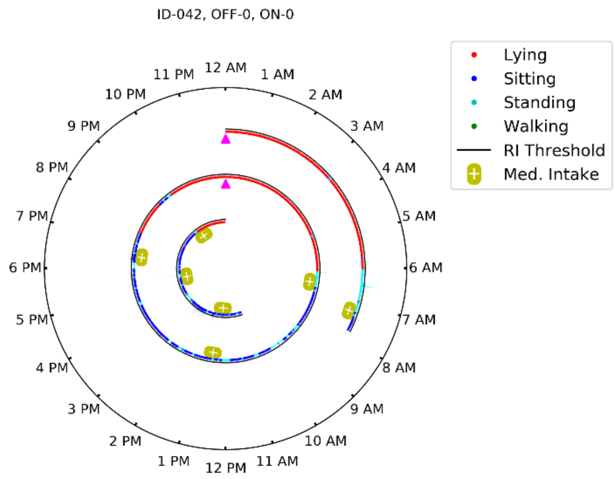
ID-042, OFF-0, ON-0
12 AM
1 AM
2 AM
3 AM
4 AM
5 AM
6 AM
7 AM
8 AM
9 AM
10 AM
11 AM
12 PM
1 PM
2 PM
3 PM
4 PM
5 PM
6 PM
7 PM
8 PM
9 PM
10 PM
11 PM
Lying
Sitting
Standing
Walking
RI Threshold
Med. Intake

(o)

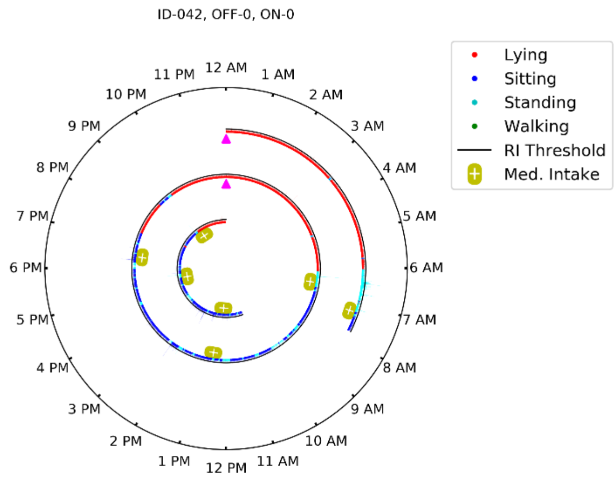
ID-042, OFF-0, ON-0
12 AM
1 AM
2 AM
3 AM
4 AM
5 AM
6 AM
7 AM
8 AM
9 AM
10 AM
11 AM
12 PM
1 PM
2 PM
3 PM
4 PM
5 PM
6 PM
7 PM
8 PM
9 PM
10 PM
11 PM
Lying
Sitting
Standing
Walking
RI Threshold
Med. Intake

(p)

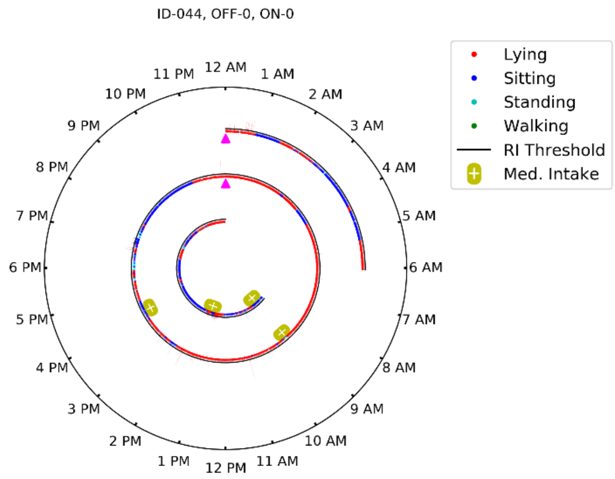

(q)

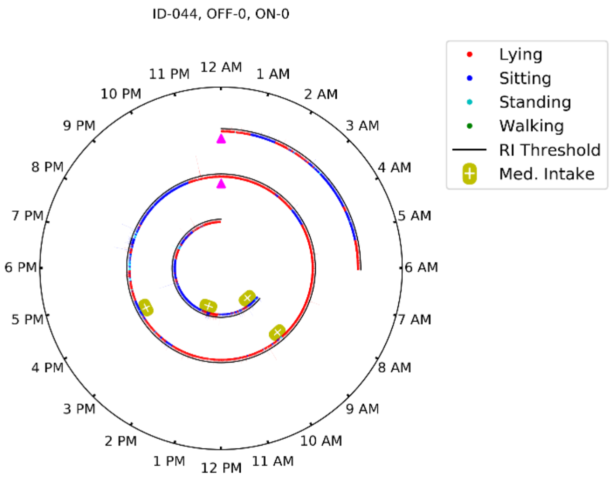

(r)

Supplementary Figure 3: Rhythmicity index profiles over full duration of the sensor wear augmented with the activity clock and illustrated for the less-affected hand of the participants with PD. Text annotations on the graph indicate the participant ID and the off/on MDS-UPDRS maximal at-rest tremor amplitude scores. OFF – NaN indicates that the participant performed only the on medication MDS-UPDRS assessments. Each color-coded bar on the polar plot jointly represents the rhythmicity index and activity state (lying, sitting, standing, or walking) over a 2-second interval, with the color identifying the activity state and the height of the bar indicating the rhythmicity index. The black circle above each radius represents the rhythmicity index threshold, which is set to a value of 3.3. The yellow capsule-shaped markers below each radius represent the medication intake times.

Supplementary Figure 4: Rhythmicity index clock visualizations for control participants

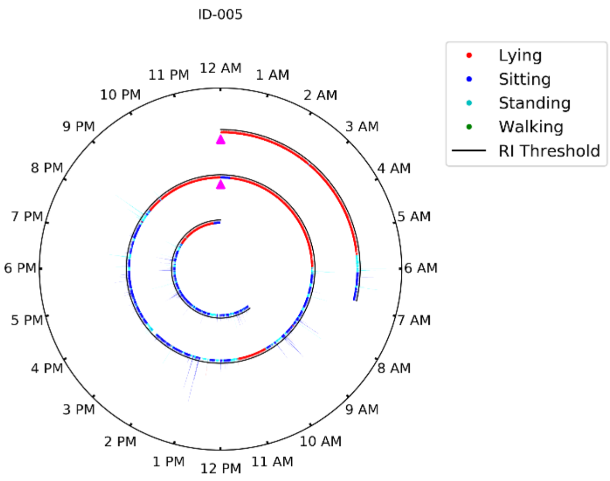

(a)

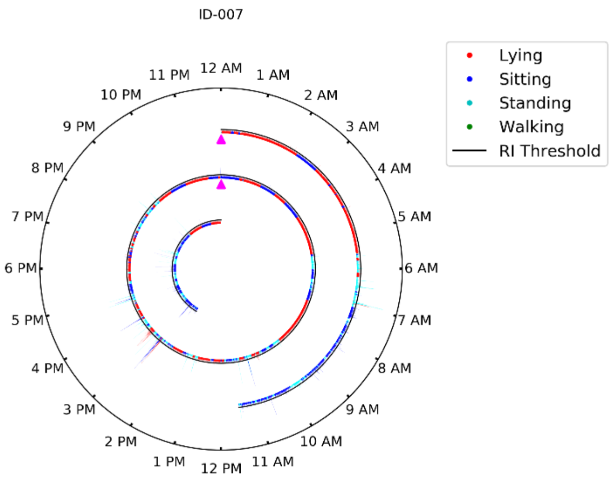

(b)

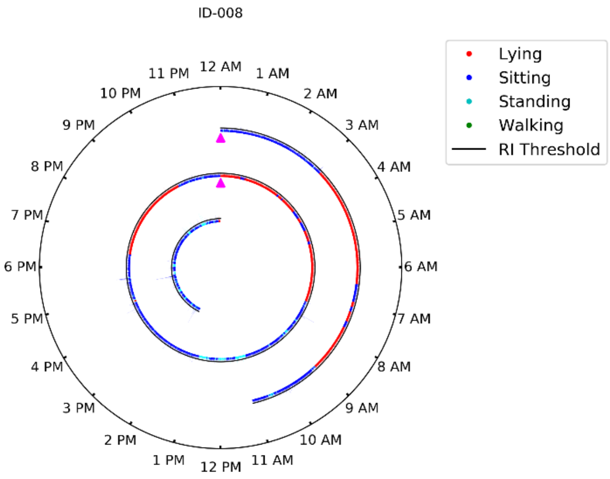

(c)

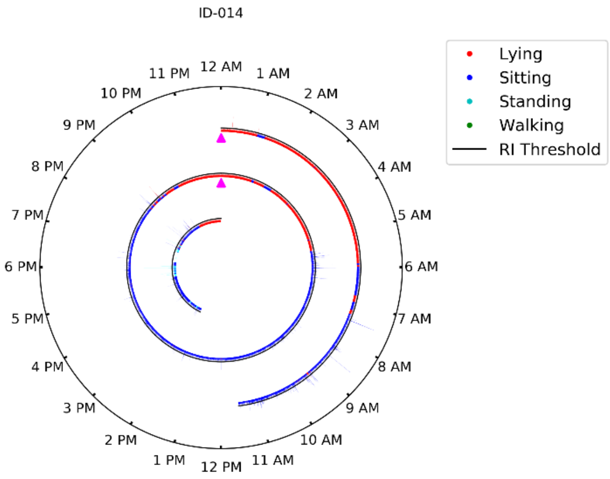

(d)

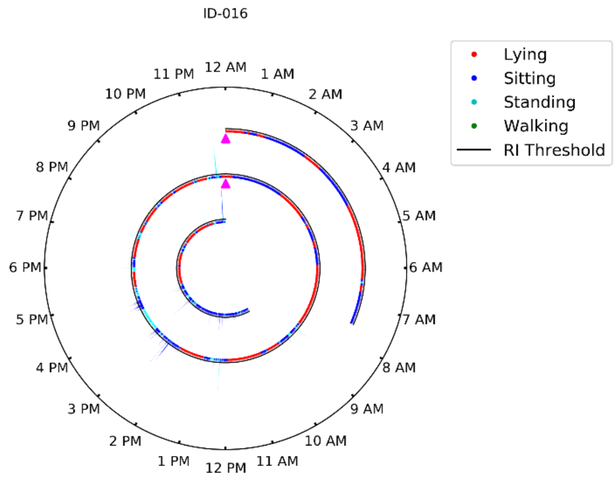
ID-016
12 AM
1 AM
2 AM
3 AM
4 AM
5 AM
6 AM
7 AM
8 AM
9 AM
10 AM
11 AM
12 PM
1 PM
2 PM
3 PM
4 PM
5 PM
6 PM
7 PM
8 PM
9 PM
10 PM
11 PM
Lying
Sitting
Standing
Walking
RI Threshold

(e)

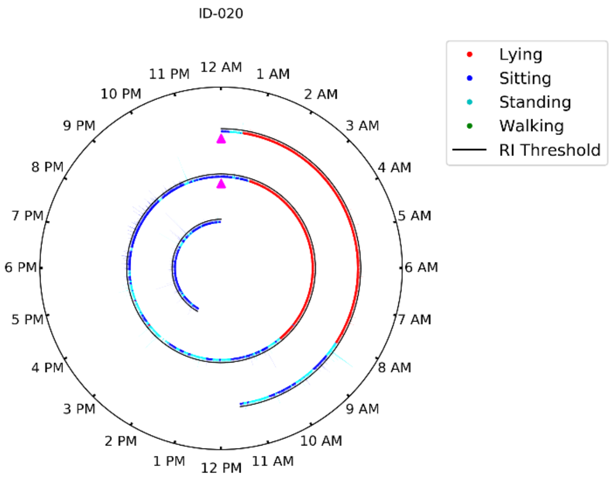
ID-020
12 AM
1 AM
2 AM
3 AM
4 AM
5 AM
6 AM
7 AM
8 AM
9 AM
10 AM
11 AM
12 PM
1 PM
2 PM
3 PM
4 PM
5 PM
6 PM
7 PM
8 PM
9 PM
10 PM
11 PM
Lying
Sitting
Standing
Walking
RI Threshold

(f)

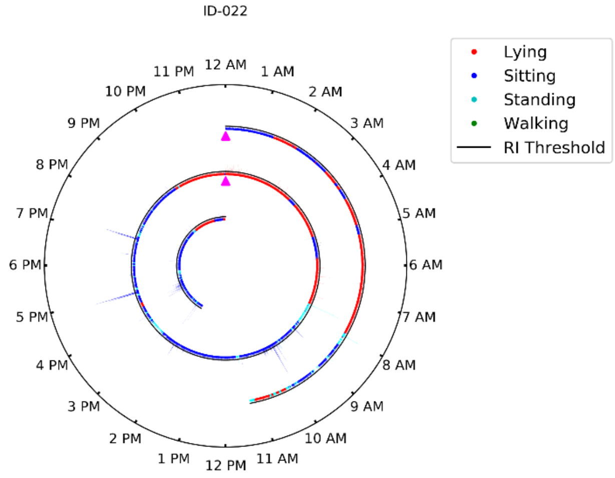
ID-022
12 AM
1 AM
2 AM
3 AM
4 AM
5 AM
6 AM
7 AM
8 AM
9 AM
10 AM
11 AM
12 PM
1 PM
2 PM
3 PM
4 PM
5 PM
6 PM
7 PM
8 PM
9 PM
10 PM
11 PM
Lying
Sitting
Standing
Walking
RI Threshold

(g)

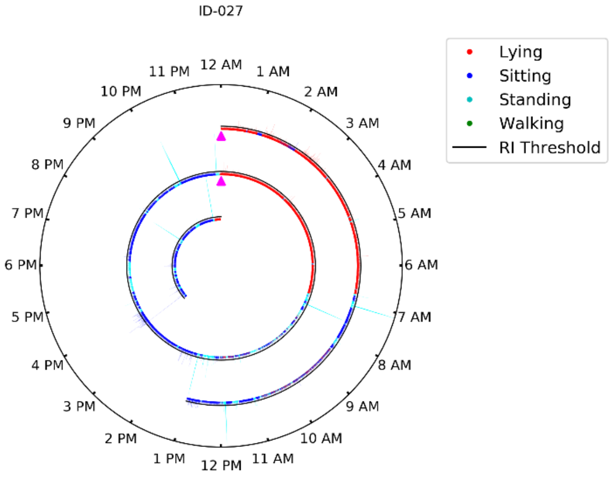
ID-027
12 AM
1 AM
2 AM
3 AM
4 AM
5 AM
6 AM
7 AM
8 AM
9 AM
10 AM
11 AM
12 PM
1 PM
2 PM
3 PM
4 PM
5 PM
6 PM
7 PM
8 PM
9 PM
10 PM
11 PM
Lying
Sitting
Standing
Walking
RI Threshold

(h)

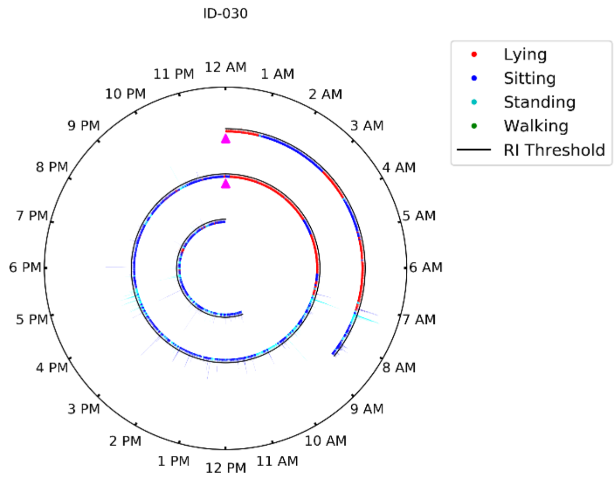
ID-030
12 AM
1 AM
2 AM
3 AM
4 AM
5 AM
6 AM
7 AM
8 AM
9 AM
10 AM
11 AM
12 PM
1 PM
2 PM
3 PM
4 PM
5 PM
6 PM
7 PM
8 PM
9 PM
10 PM
11 PM
Lying
Sitting
Standing
Walking
RI Threshold

(i)

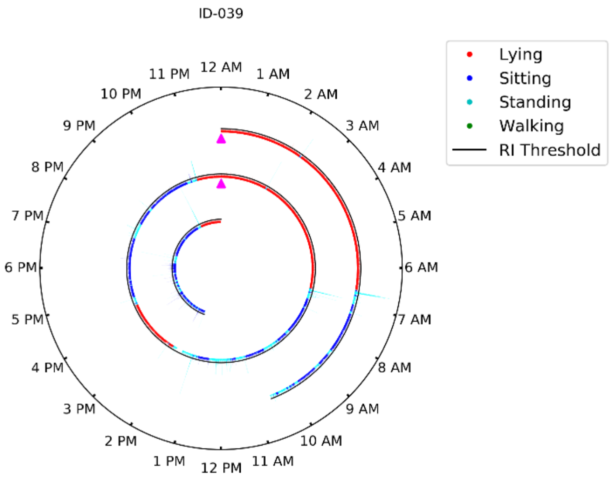
ID-039
12 AM
1 AM
2 AM
3 AM
4 AM
5 AM
6 AM
7 AM
8 AM
9 AM
10 AM
11 AM
12 PM
1 PM
2 PM
3 PM
4 PM
5 PM
6 PM
7 PM
8 PM
9 PM
10 PM
11 PM
Lying
Sitting
Standing
Walking
RI Threshold

(j)

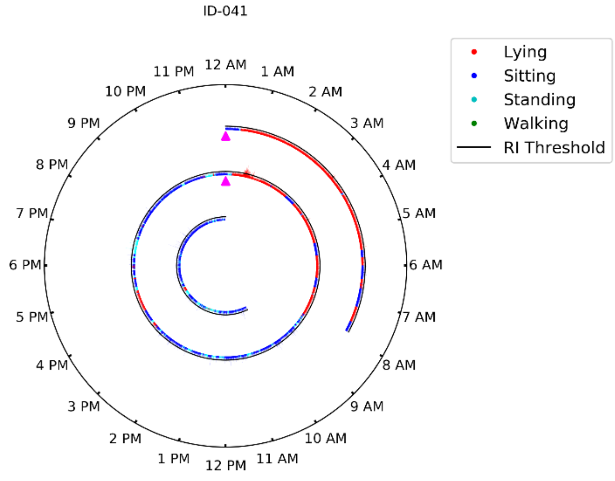
ID-041
12 AM
1 AM
2 AM
3 AM
4 AM
5 AM
6 AM
7 AM
8 AM
9 AM
10 AM
11 AM
12 PM
1 PM
2 PM
3 PM
4 PM
5 PM
6 PM
7 PM
8 PM
9 PM
10 PM
11 PM
Lying
Sitting
Standing
Walking
RI Threshold

(k)

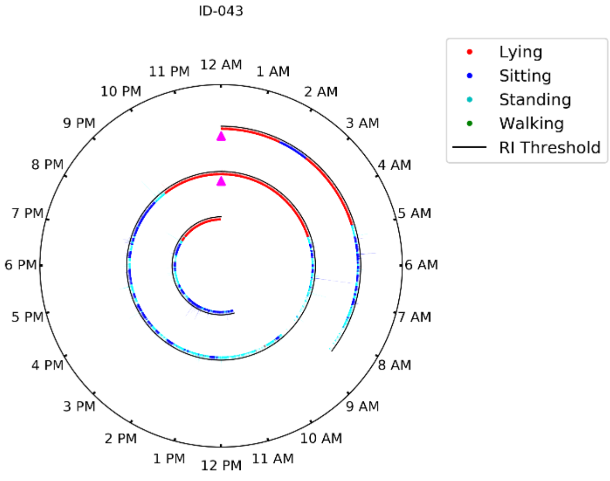
ID-043
12 AM
1 AM
2 AM
3 AM
4 AM
5 AM
6 AM
7 AM
8 AM
9 AM
10 AM
11 AM
12 PM
1 PM
2 PM
3 PM
4 PM
5 PM
6 PM
7 PM
8 PM
9 PM
10 PM
11 PM
Lying
Sitting
Standing
Walking
RI Threshold

(l)

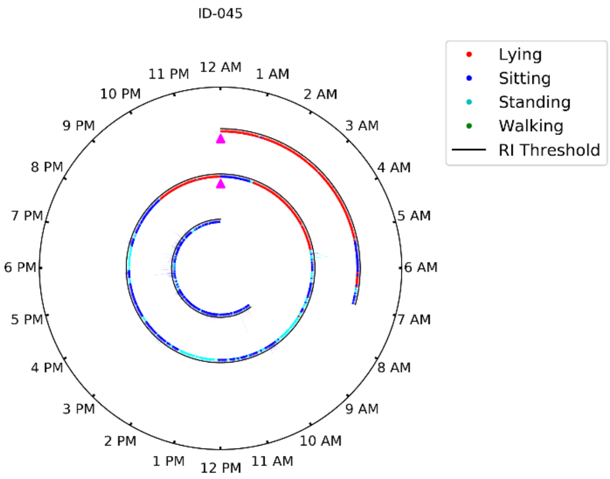

(m)

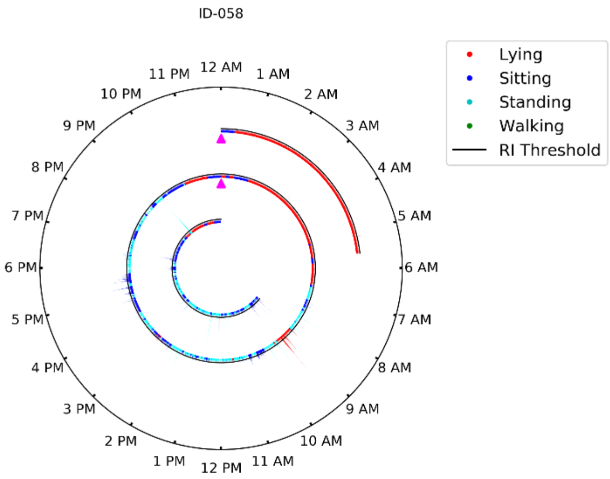

(n)

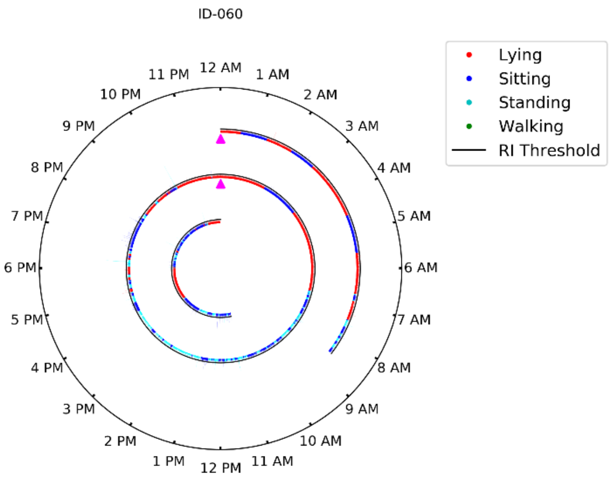

(o)

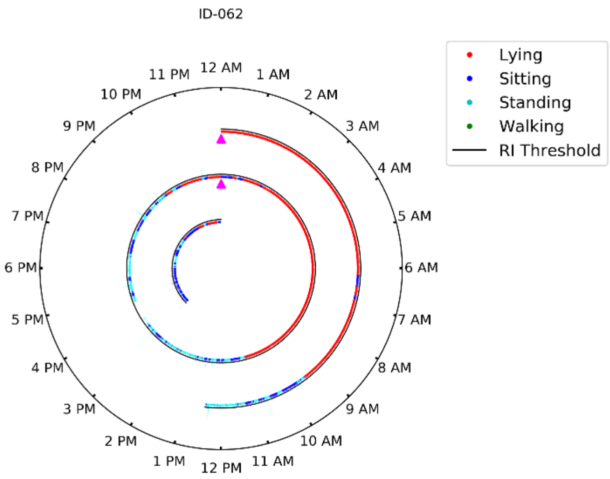

(p)

Supplementary Figure 4: Rhythmicity index profiles over full duration of the sensor wear augmented with the activity clock and illustrated for the right hand of control participants in the study. Text annotation on the graph indicates the participant ID. Each color-coded bar on the polar plot jointly represents the rhythmicity index and activity state (lying, sitting, standing, or walking) over a 2-second interval, with the color identifying the activity state and the height of the bar indicating the rhythmicity index. The black circle above each radius represents the rhythmicity index threshold, which is set to a value of 3.3.

Supplementary Figure 5: Scatter plots showing rhythmicity index and peak frequency values for the most-affected hand and less-affected hands of participants with PD and right hand of control participants over the in-clinic duration

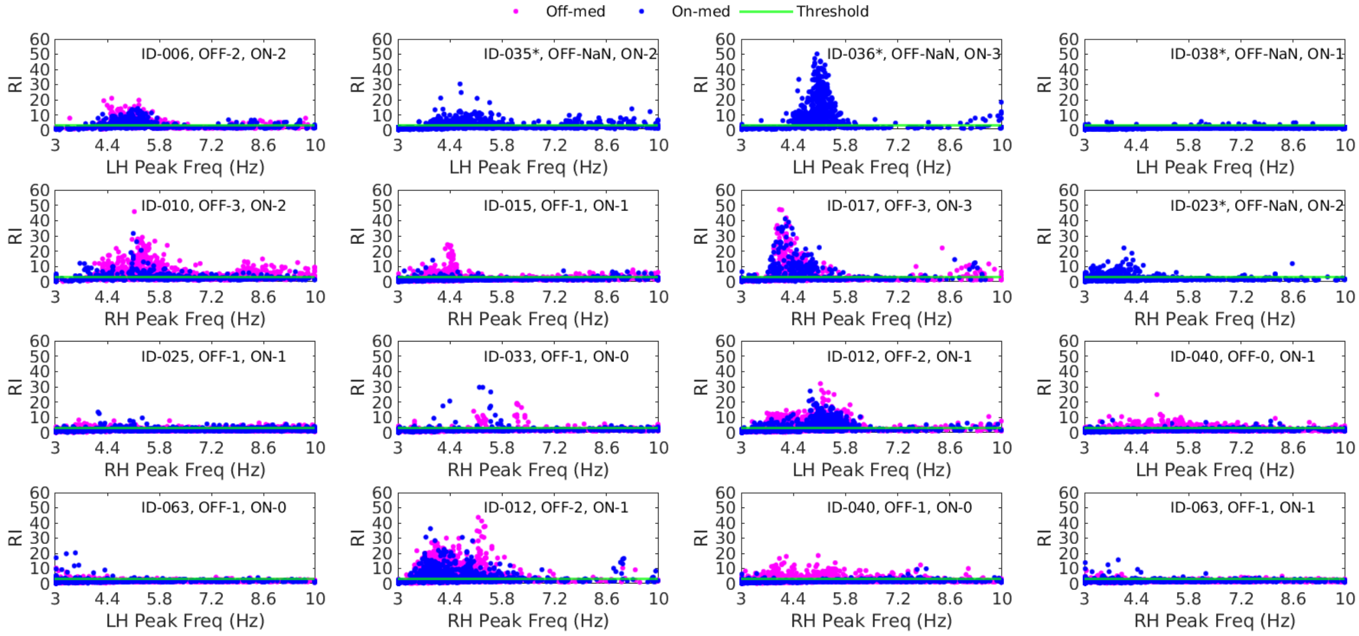

(a)

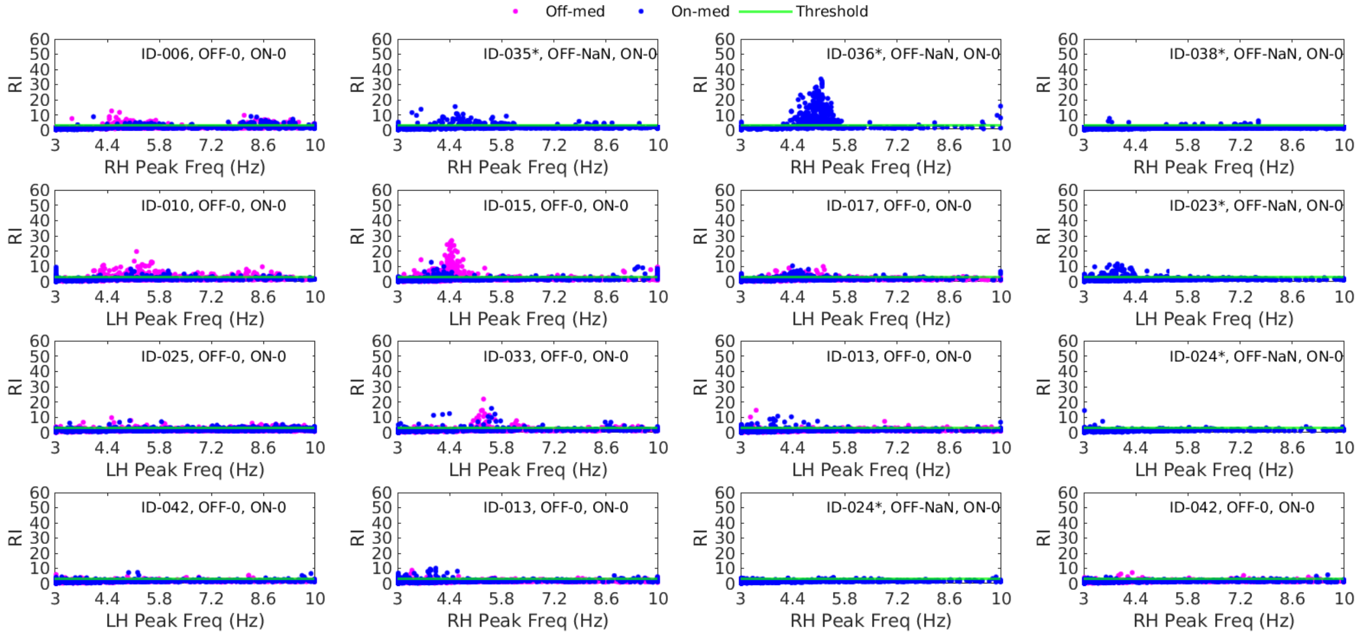

(b)

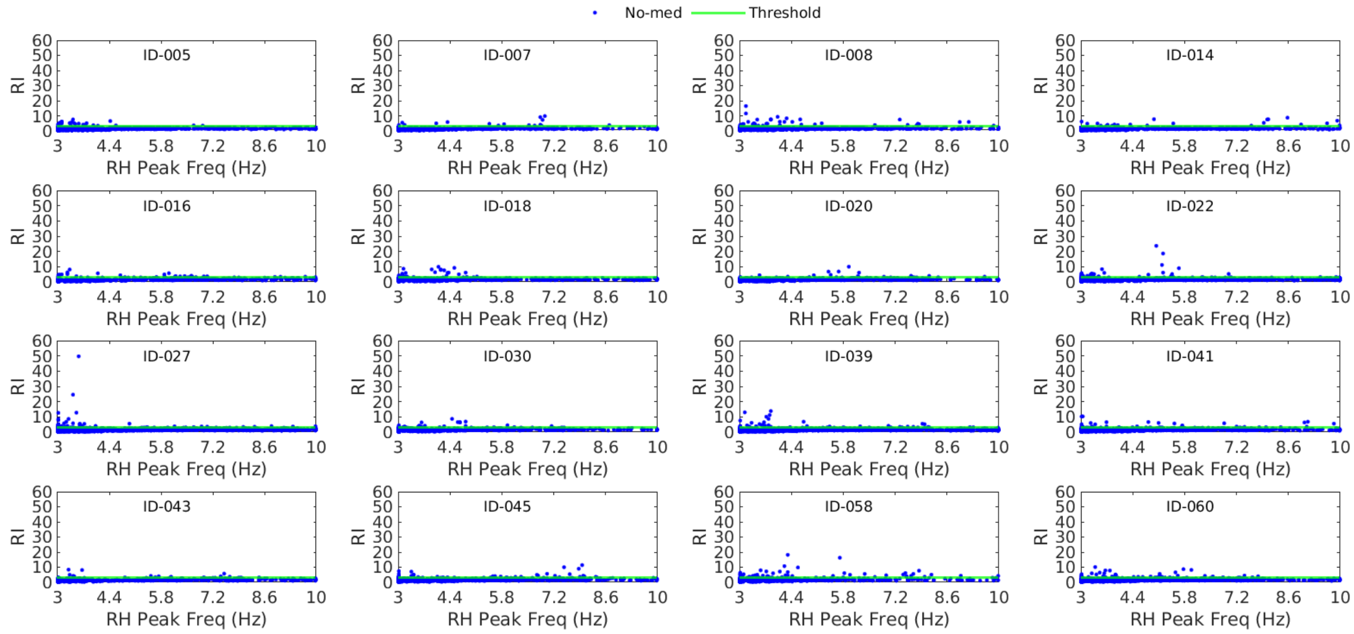

(c)

Supplementary Figure 5: Scatter plot representation showing the rhythmicity index - peak frequency values over the in-clinic durations, respectively, for the (a) most-affected and (b) less-affected hand of participants with PD, and (c) right hand of control participants. The most-affected / less-affected hand can be either right hand or left hand and is based on the in-clinic MDS-UPDRS maximal at-rest tremor scores for PD participants. Text annotations on the graph indicate the participant ID (for PD and controls) and the off/on MDS-UPDRS maximal at-rest tremor amplitude scores (only for PD participants). OFF – NaN indicates that the participant performed only the on medication MDS-UPDRS assessments. Each value in the distribution represents the estimated rhythmicity index and the corresponding peak frequency over a 2-second interval. For in-clinic durations, the distribution comprises of values both, from off (magenta) and on (blue) medication durations. The rhythmicity index threshold has a value of 3.3 and is represented by the green line. Plots are included for 16 PD participants, where 11 are on levodopa/carbidopa and 5 are on non-levodopa/carbidopa medication (indicated by *). Considerable variability in rhythmicity index and peak frequency estimates was observed both, within and across the most-affected hand of PD participants for in-clinic duration. Another noticeable trend observed for the most-affected hand of the PD participants was that the rhythmicity index during the in-clinic on-medication duration was lower in comparison with in-clinic off-medication duration.

Supplementary Figure 6: Heat maps showing rhythmicity index and peak frequency values for the most-affected and less-affected hand of participants with PD and right hand of control participants over real-world (out of clinic) durations

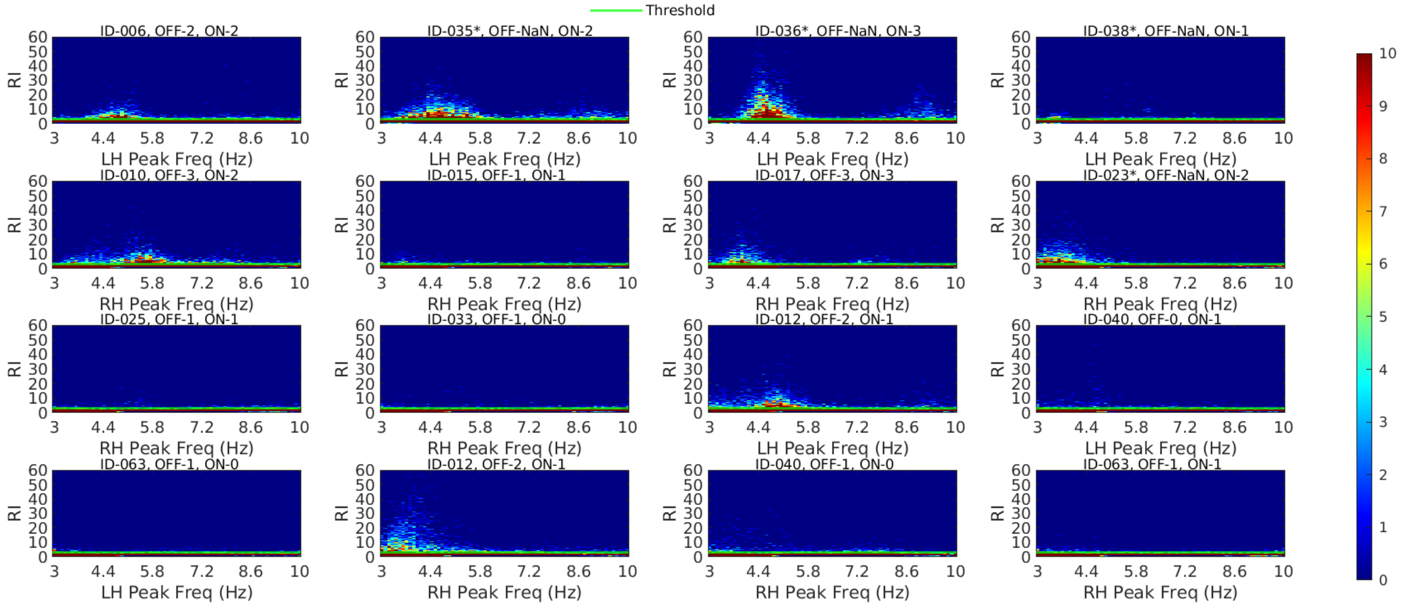

(a)

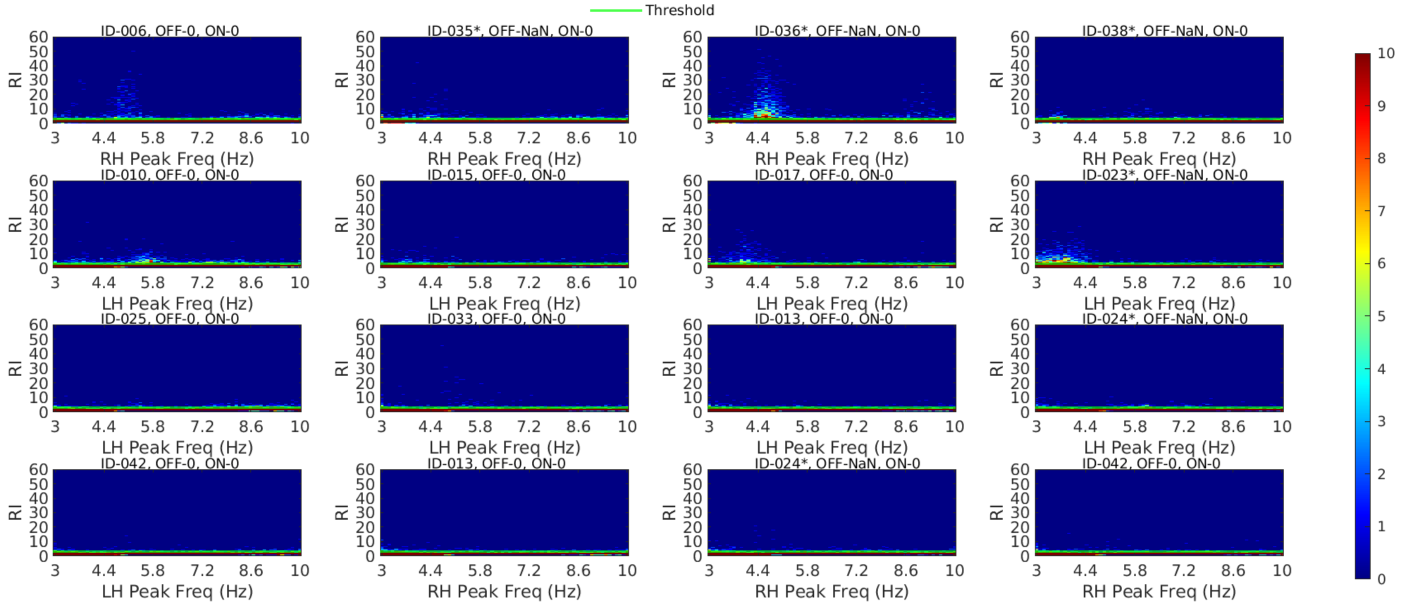

(b)

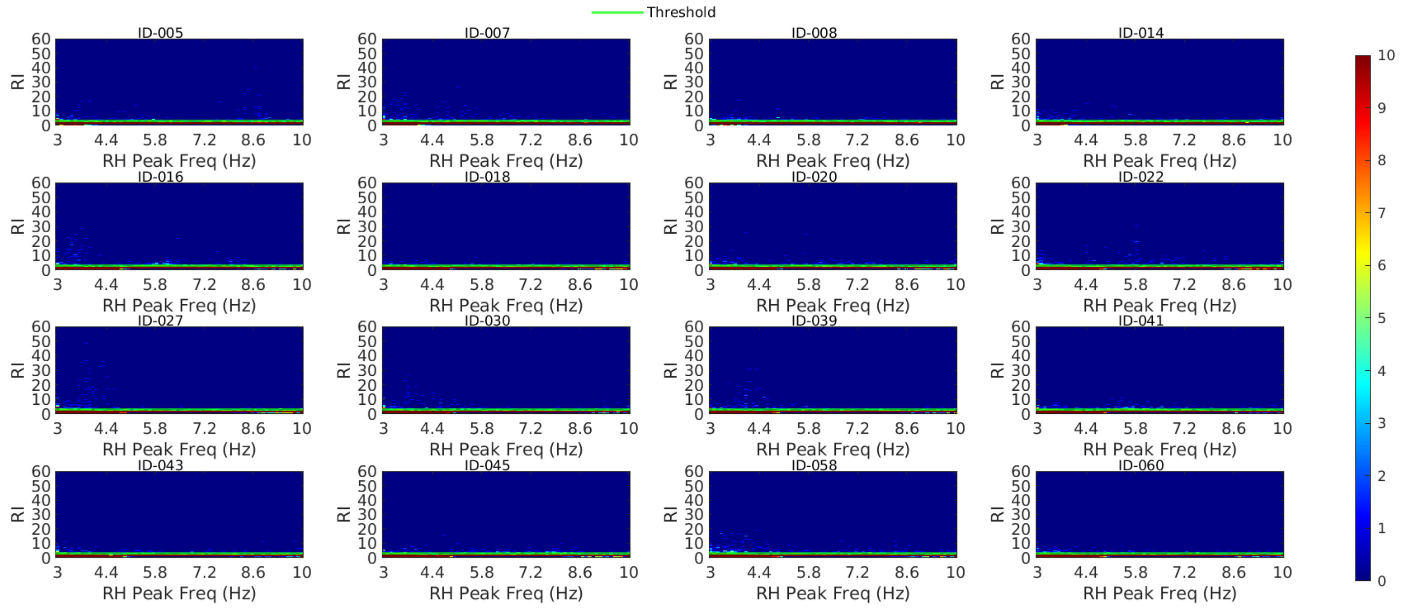

(c)

Supplementary Figure 6: Heat maps showing the rhythmicity Index - peak frequency values over the real-world duration for (a) the most-affected hand and (b) less-affected hand of participants with PD, and (c) right hand of control participants. The most-affected / less-affected hand can be either right hand or left hand and is based on the in-clinic MDS-UPDRS maximal at-rest tremor scores for participants with PD. Text annotations on the graph indicate the participant ID (for PD and controls) and the off/on MDS-UPDRS maximal at-rest tremor amplitude scores (only for PD participants). OFF – NaN indicates that the participant performed only the on medication MDS-UPDRS assessments. Each value in the distribution represents the estimated rhythmicity index and the corresponding peak frequency over a 2-second interval. The rhythmicity index threshold has a value of 3.3 and is represented by the green line. Plots are included for 16 PD participants, where 11 are on levodopa/carbidopa and 5 are on non-levodopa/carbidopa medication (represented by *) and 16 controls. For the real-world duration, the rhythmicity index / peak frequency trends for the most-affected hand of participants with PD were analogous to those observed during the in-clinic duration.

Supplementary Table 1: An example of activity log used in the study

Date	Start Time	End Time	Activity	Description/Note
6/1/2020	10:30 pm	6:00 am	Sleeping	Got up at 3 am to use bathroom
6/2/2020	7 am	N/A	Took meds	2 tablets of carbidopa/levodopa
6/2/2020	9 am	10 am	Running	Sensors fell off at 9:45 am; put it back on immediately
