# Supplementary material for: A real-world study of wearable sensors in Parkinson’s disease
Source: NPJ Parkinsons Dis. 2021 Nov 29;7:106. doi: 10.1038/s41531-021-00248-w (PMC8629990; doi:10.1038/s41531-021-00248-w)
Supplement: Supplementary file 2 — Reporting Summary [file 41531_2021_248_MOESM2_ESM.pdf]

nature portfolio

Corresponding author(s): Jamie Adams

Last updated by author(s): Aug 18, 2021

Reporting Summary

Nature Portfolio wishes to improve the reproducibility of the work that we publish. This form provides structure for consistency and transparency in reporting. For further information on Nature Portfolio policies, see our Editorial Policies and the Editorial Policy Checklist.

Statistics

For all statistical analyses, confirm that the following items are present in the figure legend, table legend, main text, or Methods section.

n/a	Confirmed	
☐	☒	The exact sample size (*n*) for each experimental group/condition, given as a discrete number and unit of measurement
☐	☒	A statement on whether measurements were taken from distinct samples or whether the same sample was measured repeatedly
☐	☒	The statistical test(s) used AND whether they are one- or two-sided<br>*Only common tests should be described solely by name; describe more complex techniques in the Methods section.*
☐	☒	A description of all covariates tested
☐	☒	A description of any assumptions or corrections, such as tests of normality and adjustment for multiple comparisons
☐	☒	A full description of the statistical parameters including central tendency (e.g. means) or other basic estimates (e.g. regression coefficient) AND variation (e.g. standard deviation) or associated estimates of uncertainty (e.g. confidence intervals)
☐	☒	For null hypothesis testing, the test statistic (e.g. *F*, *t*, *r*) with confidence intervals, effect sizes, degrees of freedom and *P* value noted<br>*Give P values as exact values whenever suitable.*
☒	☐	For Bayesian analysis, information on the choice of priors and Markov chain Monte Carlo settings
☒	☐	For hierarchical and complex designs, identification of the appropriate level for tests and full reporting of outcomes
☒	☐	Estimates of effect sizes (e.g. Cohen's *d*, Pearson's *r*), indicating how they were calculated

Our web collection on statistics for biologists contains articles on many of the points above.

Software and code

Policy information about availability of computer code

Data collection	Accelerometry data from wearable sensors was collected using the MC 10 system (https://www.mc10inc.com/) which provided access to the recorded data via a web-portal.
Data analysis	Data was analyzed using MATLAB® (version 2019b, MathWorks, Natick, MA) and visualization plots were generated using Python 3.7.4.

For manuscripts utilizing custom algorithms or software that are central to the research but not yet described in published literature, software must be made available to editors and reviewers. We strongly encourage code deposition in a community repository (e.g. GitHub). See the Nature Portfolio guidelines for submitting code & software for further information.

Data

Policy information about availability of data

All manuscripts must include a data availability statement. This statement should provide the following information, where applicable:

- Accession codes, unique identifiers, or web links for publicly available datasets
- A description of any restrictions on data availability
- For clinical datasets or third party data, please ensure that the statement adheres to our policy

The sensor accelerometry and MDS-UPDRS assessment-task annotation data for each participant, and demographic and clinical assessment data for all participants will be made available at IEEE DataPort with identifier “doi: dx.doi.org/10.21227/g2g8-1503”. Currently, sample accelerometry and MDS-UPDRS assessment-task annotation data for one participant is provided; with the publication of the paper, data for all the participants will be made available in the same repository.

Field-specific reporting

Please select the one below that is the best fit for your research. If you are not sure, read the appropriate sections before making your selection.

☒ Life sciences ☐ Behavioural & social sciences ☐ Ecological, evolutionary & environmental sciences

For a reference copy of the document with all sections, see nature.com/documents/nr-reporting-summary-flat.pdf

Life sciences study design

All studies must disclose on these points even when the disclosure is negative.

Sample size	Since we conducted a pilot study to analyze the tremor, activity, and gait characteristics of PD in both clinic and real-world using wearable sensors, we chose a small sample size (20 PD and 22 controls).
Data exclusions	Twenty individuals with PD and 22 controls were enrolled in the study. Three individuals with PD were excluded from analysis due to sensor problems. Five controls were excluded from analysis after age-matching participants with PD to the controls. Data from 17 PD and 17 control participants were used for analysis
Replication	Our tremor algorithm will be further refined and tested in a current study that we are enrolling for. As noted, our gait and activity analyses have been used in other cohorts of this study and this data has been published.
Randomization	For tremor analysis, since different hands exhibited different range of tremor amplitude and frequency, we analyzed “most-affected” and “less-affected” hands separately. A hand was considered “most-affected” if the MDS-UPDRS maximal at-rest tremor score (MDS-UPDRS 3.17a - 3.17b) for the hand ranged from 1 to 4 and “less-affected” hands were identified by a MDS-UPDRS maximal at-rest tremor score of 0. For activity and gait analysis, the grouping was straight-forward as we wanted to compare PD vs the controls.
Blinding	The blinding was not necessary as this was an observational study, where our goal was to examine the activity profile and subsequently analyze the gait and tremor characteristics of participants in the clinic and real-world using wearable sensors.

Reporting for specific materials, systems and methods

We require information from authors about some types of materials, experimental systems and methods used in many studies. Here, indicate whether each material, system or method listed is relevant to your study. If you are not sure if a list item applies to your research, read the appropriate section before selecting a response.

Materials & experimental systems

n/a	Involved in the study	
☒	☐	Antibodies
☒	☐	Eukaryotic cell lines
☒	☐	Palaeontology and archaeology
☒	☐	Animals and other organisms
☐	☒	Human research participants
☐	☒	Clinical data
☒	☐	Dual use research of concern

Methods

n/a	Involved in the study	
☒	☐	ChIP-seq
☒	☐	Flow cytometry
☒	☐	MRI-based neuroimaging

Human research participants

Policy information about studies involving human research participants

Population characteristics	Twenty individuals with PD and 22 controls were enrolled in the study. Some participants were excluded from the analysis for the reasons described above. Data from 17 PD (mean [standard deviation] age: 66.4 [11.3] years; 41.2% women) and 17 control (64.0 [9.9] years; 76.5% women) participants were used for analysis. Of the 17 PD participants, 8 were classified as postural instability/gait difficulty (PIGD), 7 as tremor dominant (TD), and 2 as indeterminate motor phenotypes.
Recruitment	We recruited individuals with PD from clinics, study interest registries, and regional support groups. Control participants were comprised of unaffected spouses, family members, friends, and community members.
Ethics oversight	The University of Rochester’s institutional review board

Note that full information on the approval of the study protocol must also be provided in the manuscript.

Clinical data

Policy information about clinical studies

All manuscripts should comply with the ICMJE guidelines for publication of clinical research and a completed CONSORT checklist must be included with all submissions.

Clinical trial registration	This is an observational pilot study and was not registered in clinicaltrials.gov.
Study protocol	The study protocol can be made available by contacting the corresponding author.
Data collection	Participants were enrolled between June 2016 and April 2017. Study visits were conducted at the University of Rochester, Movement Disorders Clinic. Accelerometry data from wearable sensors was collected using the MC 10 system (https://www.mc10inc.com/) which provided access to the recorded data via a web-portal.
Outcomes	Tremor proportion was the primary outcome measure. We compared the tremor proportion between the most-affected and less-affected hands of the PD participants and right hand of control participants. We also classified our PD cohort in to PIGD and TD motor phenotypes and compared the most-affected hand tremor proportion. Finally we also performed correlation analysis to assess relation between the derived tremor proportion and clinical scores. The proportion of time spent in different activities and gait parameters were the secondary outcome measures, which were compared between the PD and controls.
